# A Comprehensive Study of Vesicular and Non-Vesicular miRNAs from a Volume of Cerebrospinal Fluid Compatible with Clinical Practice

**DOI:** 10.7150/thno.31502

**Published:** 2019-06-19

**Authors:** Endika Prieto-Fernández, Ana María Aransay, Félix Royo, Esperanza González, Juan José Lozano, Borja Santos-Zorrozua, Nuria Macias-Camara, Monika González, Raquel Pérez Garay, Javier Benito, Africa Garcia-Orad, Juan Manuel Falcón-Pérez

**Affiliations:** 1 Department of Genetics, Physical Anthropology and Animal Physiology, Faculty of Medicine and Nursing, University of The Basque Country (UPV/EHU), Leioa, Bizkaia, 48940, Spain.; 2 Genome Analysis Platform, CIC bioGUNE, Derio, Bizkaia, 48980, Spain.; 3 Centro de Investigación Biomédica en Red de Enfermedades Hepáticas y Digestivas (CIBERehd), Instituto de Salud Carlos III, Madrid, 28029, Spain.; 4 Exosomes Lab, CIC bioGUNE, CIBERehd, Derio, Bizkaia, 48980, Spain.; 5 Bioinformatics Unit, Centre Esther Koplovitz (CEK), CIBERehd, Barcelona, 08036, Spain.; 6 Biochemistry Service, Cruces University Hospital, Barakaldo, Bizkaia, 48903, Spain.; 7 Department of Pediatric Emergency, Cruces University Hospital, Barakaldo, Bizkaia, 48903, Spain.; 8 Department of Pediatrics, University of The Basque Country (UPV/EHU), Leioa, Bizkaia, 48940, Spain.; 9 BioCruces Health Research Institute, Barakaldo, Bizkaia, 48903, Spain.; 10 IKERBASQUE, Basque Foundation for Science, Bilbao, Bizkaia, 48015, Spain.

**Keywords:** CSF miRNAs, CSF exosomes, microRNA profiling, infants, clinical samples

## Abstract

Cerebrospinal fluid (CSF) microRNAs (miRNAs) have emerged as potential biomarkers for minimally invasive diagnosis of central nervous system malignancies. However, despite significant advances in recent years, this field still suffers from poor data reproducibility. This is especially true in cases of infants, considered a new subject group. Implementing efficient methods to study miRNAs from clinically realistic CSF volumes is necessary for the identification of new biomarkers.

**Methods**: We compared six protocols for characterizing miRNAs, using 200-µL CSF from infants (aged 0-7). Four of the methods employed extracellular vesicle (EV) enrichment step and the other two obtained the miRNAs directly from cleared CSF. The efficiency of each method was assessed using real-time PCR and small RNA sequencing. We also determined the distribution of miRNAs among different CSF shuttles, using size-exclusion chromatography.

**Results**: We identified 281 CSF miRNAs from infants. We demonstrated that the miRNAs could be efficiently detected using only 200 µL of biofluid in case of at least two of the six methods. In the exosomal fraction, we found 12 miRNAs that might be involved in neurodevelopment.

**Conclusion**: The Norgen and Invitrogen protocols appear suitable for the analysis of a large number of miRNAs using small CSF samples.

## Introduction

Cerebrospinal fluid (CSF) is a potential source for minimally invasive diagnostic analysis of neurological disorders, including viral infections 1, Alzheimer's disease 2, 3, traumatic brain injury 4, and brain tumors 5-7. The CSF contains cells, extracellular vesicles (EVs), and biomolecules such as proteins, nucleic acids, and metabolites. These biomolecules can be either associated with the cells or EVs 3, 4 or circulate freely within the fluid 8. Among these components, the microRNAs (miRNAs) have been attracting increasing attention in recent years 8, 9. miRNAs are short (∼22 nucleotides) non-coding RNAs that modulate gene expression at the post-transcriptional level 10. They regulate more than 50% of human genes, including many that are related to cancer 11, 12. Some changes in the levels of certain miRNAs have already been associated with various pathologies 8. Teplyuk *et al.* have found high levels of miR-10b and miR-21 in the CSF of patients with glioblastoma (GBM). Interestingly, the increased levels of these two miRNAs are also associated with the metastasis to the brain in patients with primary breast and lung cancers 13. Baraniskin *et al.* have reported that miR-21 and miR-15b are upregulated in CSF samples from patients with glioma 14.

However, these studies analyzed CSF miRNAs without examining their transport in the fluid. The distribution of miRNAs among miRNA shuttles in the CSF, i.e., proteins, lipoproteins, and EVs (exosomes, microvesicles, and apoptotic bodies), can differ between normal and pathological conditions 8. Yagi *et al.* have recently observed that the CSF miRNAs can vary between the vesicular and non-vesicular fractions. They have shown that the miRNAs associated with EVs are different from those found in free circulation. miR-1911-5p, miR-1264, and miR-34b, among others, are abundant in CSF EVs and not in the EV-depleted CSF 15. EVs are released by all types of cells and can enter the bodily fluids 16. Cancer cells also actively release EVs carrying miRNAs, which communicate with near and distal cells in the tumor microenvironment and affect tumor progression 17, 18, contributing to the final vesicular composition of biofluids. Therefore, miRNAs carried by CSF EVs might constitute a good source of biomarkers for minimally invasive diagnosis and prognosis of brain cancer. Although the CSF is often obtained for diagnosis in the clinical practice, the EVs that this fluid contains are not routinely examined, neglecting most of its biomarker potential. Only one study has currently reported a robust CSF EV-associated miRNA signature (nine-miRNA catalog) for minimally invasive diagnosis of GBM 19.

Despite significant advances in recent years, this field suffers from poor data reproducibility 20, 21. The main reason for this persistent problem is the complexity of CSF miRNA detection procedures, with many unresolved technical issues 8. First, it is crucial to preserve the integrity of CSF specimens between their collection and laboratory processing and storage. The samples should be processed within 2 hours of collection, and freeze-thaw cycles should be avoided 20. Second, various existing miRNA isolation protocols have different yields. To date, various methods and kits have been used to isolate EVs from CSF samples 15, 19, 22-28 and to extract miRNAs from CSF EVs 15, 19, 22-27, 29 and from cleared CSF 9, 13, 14, 30-39. However, it is difficult to compare the data obtained using such a varying array of techniques, kits, and samples. Third, different miRNA profiling platforms and data normalization approaches can result in distinct miRNA catalogs 20, 36. In view of the above, the unresolved technical issues in the analysis and standardization of protocols are still the obstacles to be overcome. Another important problem to be considered is the limited amount of CSF that can be obtained for the diagnosis. In the case of pediatric patients, this problem is even more acute since the amount of CSF routinely withdrawn for clinical tests is smaller than from the adults. For this reason, these patients constitute a largely unexplored subject group for which new biomarkers are desperately needed 21. In addition, very little is known of the biological variability of the CSF microRNAome; it might be affected by the diet, health status, age, and development. Establishing a standardized, efficient protocol for miRNA detection in small CSF samples would facilitate the necessary large studies with statistically sufficient numbers of subjects. The availability of such methods might also help to clarify other issues and identify new biomarkers, especially in infants, where they are desperately needed 21.

Thus, it is necessary to adapt the existing protocols to small CSF sample volumes and find the most appropriate method for miRNA analysis. Here, we performed a comprehensive comparative analysis of six existing protocols to establish a simple and effective method for detecting miRNAs from a minimum CSF volume of clinical specimens. We placed particular emphasis on identifying miRNAs associated with EVs. We believe that our study contributes some valuable data to the search of new low-invasive biomarkers for pediatric brain cancers and to their implementation in clinical practice.

## Methods

### Experimental design

In this study, we performed a comprehensive comparative analysis of six existing methods to define a simple and effective strategy for detecting miRNAs from 200-µL samples of CSF. Four of the protocols included an initial EVs enrichment step. The other two were designed to extract miRNAs directly from cleared CSF. First, the efficiency of each method was assessed using the real-time PCR (RT-qPCR). Eight miRNAs previously detected in the CSF by Yagi *et al.* were selected as reference 15. We used small RNA sequencing (smallRNAseq) data from Yagi *et al.* as a reference throughout our study since they isolated miRNAs from a large volume of CSF (7 mL). The eight miRNAs had variable concentrations in the exosomal fractions of Yagi *et al.* (Figure S1A) 15. Our aim was to establish the detection limit of each of the methods for small CSF volumes (200 µL). Then, the methods capable of identifying the majority of the eight miRNAs (using RT-qPCR) were examined employing the smallRNAseq to find the technique detecting the largest number of miRNAs.

**Figure 1:**
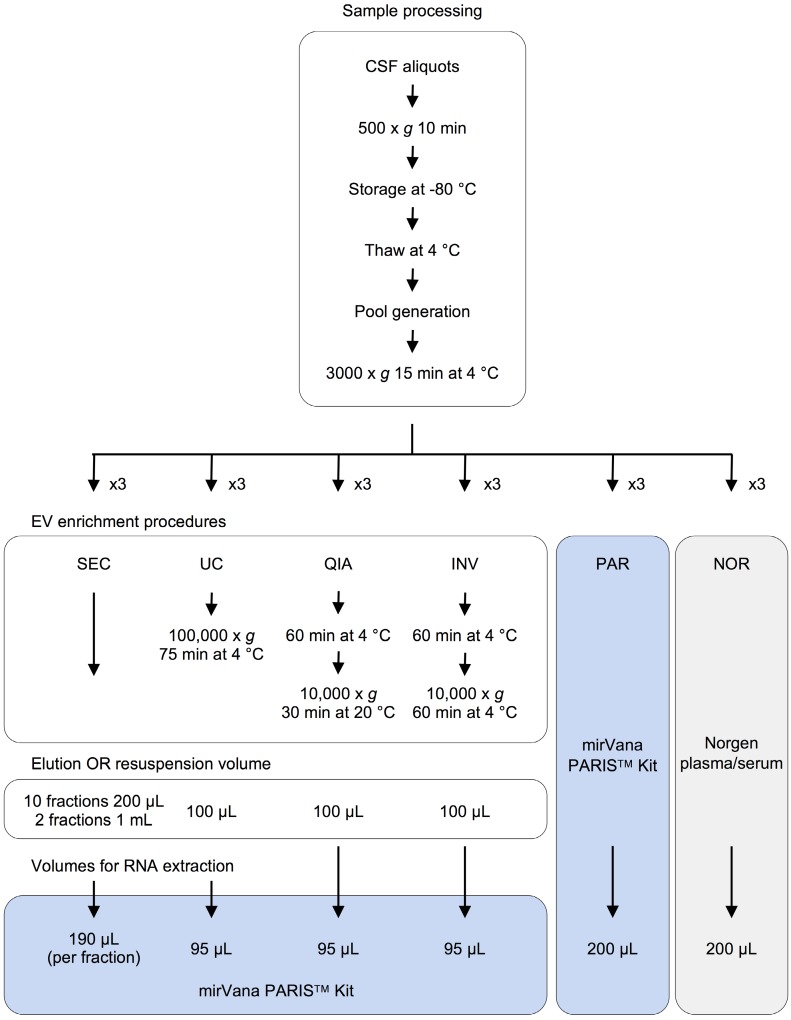
Experimental design for comparison of six methods of analyzing miRNAs in bodily fluids. Aliquots of 200 µL of CSF were used to test each of the six methods in triplicate (overall, 18 aliquots were processed). The pellets obtained in each EV enrichment procedure were resuspended in 100 µL of 1X DPBS. Then, total RNA was extracted to perform the downstream analyses (TaqMan RT-qPCR and smallRNAseq). Abbreviations: size-exclusion chromatography (SEC), ultracentrifugation (UC), miRCURY Exosome Isolation Kit from Qiagen (QIA), Total Exosome Isolation Reagent from Invitrogen (INV), mirVana PARIS Kit from Ambion (PAR), and Plasma/Serum RNA Purification Kit from Norgen (NOR).

Moreover, the CSF samples were fractionated using an in-house size-exclusion chromatography (SEC) method. Two fractions representing the exosomal and supernatant fractions were sequenced to identify enriched miRNAs in the exosomal fraction of CSF of infants. Each procedure was carried out in triplicate (Figure 1).

### Human CSF samples

Samples and data from patients included in this study were provided by the Basque Biobank (www.biobancovasco.org) and were processed following standard operation procedures with appropriate approval of the Ethical and Scientific Committees (code CEIC E17/40). Nineteen non-hemorrhagic samples from children (aged 0-7) were acquired. The CSF samples were obtained via lumbar puncture, centrifuged to remove contaminant cells (500 x *g* for 10 minutes), aliquoted, and immediately stored at -80 °C until processing. To perform the main experiment and the analyses of reproducibility and RNase protection, three independent pools of samples were generated. To achieve that, the CSF samples were thawed at 4 °C, mixed together, centrifuged at 3,000 x *g* for 15 minutes at 4 °C, divided into 200 µL-aliquots (cleared CSF), and kept on ice until processing.

### EVs enrichment procedures

Four different EV enrichment methods were evaluated in triplicate: ultracentrifugation (UC), miRCURY Exosome Isolation Kit - Cells, Urine and CSF (Qiagen #76743) (QIA), Total Exosome Isolation Reagent (Invitrogen #4484453) (INV), and an in-house SEC. UC was carried out in a single step (100,000 x *g* for 75 minutes at 4 °C) using a Beckman-Coulter TLA 120.2 rotor. The QIA kit was used following the manufacturer's instructions. The INV method was slightly modified; the CSF triplicates were not initially centrifuged at 10,000 x *g*, as recommended, but at 3000 x *g* like in the rest of the protocols*.* The UC and QIA methods required an adjustment of the sample volume from 200 µL to 1.0 mL using 1X DPBS (Gibco #14190-094). In all cases, the EV pellets were resuspended in 100 µL of 1X DPBS. Finally, the cleared CSF was fractionated using the in-house SEC. This was performed as follows: The Poly-prep Chromatography Column (BioRad #731-1550) was filled with 2.5 mL of Sepharose CL-2B cross-linked resin (Sigma #CL2B300-100ML) and left packing overnight at 4 °C. Then, the column was washed twice with 2.5 ml of 1X DPBS. Once the sample (200 µL of CSF) was applied onto the column, 4.0 mL of 1X DPBS was added; 10 fractions of 200 µL and two final fractions of 1.0 mL were collected. The RNA was extracted from the isolated EVs and the SEC fractions using the mirVana PARIS Kit (Ambion #AM1556) (PAR), following the manufacturer's instructions for total RNA isolation. To account for the differences in the efficiency of the extraction, the samples were spiked with cel-miR-39 (2x10^-4^ nmoles added) (Invitrogen) after mixing the cleared CSF with the cell disruption buffer (Figure 1).

### RNA extraction directly from cleared CSF

Total RNA was extracted from 200 µL of cleared CSF using PAR and the Plasma/Serum RNA Purification Kit (Norgen #55000) (NOR). The isolated RNA was eluted in 100 µL of nuclease‑free water (Ambion #AM9930). Before each RNA extraction, the samples were spiked with cel-miR-39 (2x10^-4^ nmoles added) (Invitrogen) (Figure 1).

### cDNA synthesis

The cDNA was synthesized from 2 µL of the isolated RNA using the TaqMan Advanced miRNA cDNA Synthesis Kit (Applied Biosystems #A28007), following the manufacturer's recommendations. To account for differences in the retrotranscription reaction and for the calculation of relative quantities of each miRNA, 2x10^-8^ nmoles of ath-miR-159a (Invitrogen) were added to each reaction.

### Real-Time qPCR assay

The reaction mix consisted of 5 µL of TaqMan Fast Advanced Master Mix (Applied Biosystems #4444557), 0.5 µL of TaqMan Advanced miRNA Assays (Applied Biosystems #A25576), 1.5 µL of nuclease-free water (Ambion #AM9930), and 3 µL of cDNA diluted at 1:3 ratio. The following assays were performed: ath-miR-159a (478411_mir) and cel-miR-39-3p (478293_mir) for detecting the spike-ins, as well as hsa-miR-21-5p (477975_mir), hsa-miR-451a (478107_mir), hsa-miR-92a-3p (477827_mir), hsa-miR-22-3p (477985_mir), hsa-miR-1911-5p (479583_mir), has-miR-1264 (478670_mir), hsa-miR-30c-5p (478008_mir), and hsa-miR-34b-3p (478049_mir) (Applied Biosystems) to detect the eight miRNAs selected. The RT-qPCR reactions were conducted in duplicate on a ViiA 7 Real-Time PCR System (Applied Biosystems). The data were analyzed using the QuantStudio Real-Time PCR System, software version 1.3 (Applied Biosystems).

### Western blot analysis of SEC fractions

First, 200 µL-aliquot of CSF was fractionated using SEC as previously described. Then, 150 µL of each fraction was concentrated using 99.5% acetone (Panreac #161007) and resuspended in 20 µL of 1X DPBS. Fifteen µL of the suspension was mixed with 5 µL of NuPAGE LDS Sample Buffer 4X (Invitrogen #NP0007) and heated for 5 minutes at 37 °C, 10 minutes at 65 °C, and 15 minutes at 95 °C. Each preparation was separated in a 4-12% Bis-Tris gel (Invitrogen #NP0336BOX) with MOPS SDS Running Buffer 20X (Invitrogen #NP0001). Precision Plus Protein Dual Color Standard (BioRad #161-0374) was used to calculate the molecular weights of the proteins. The proteins were transferred to an Immobilon-P Transfer membrane (Merck Millipore #IPVH00010) using the NuPAGE Transfer Buffer 20X (Invitrogen #NP0006-1) and blocked for 1 hour in 5% Blotting-Grade Blocker (BioRad #170-6404) and 0.2% Tween-20 (Sigma Aldrich #P2287) diluted in 1X DPBS. Then, the primary antibodies (1:500) were added and incubated overnight, followed by three washes with 1X DPBS and the application of secondary HRP-conjugated antibodies (1:6000). Primary antibodies against exosomal markers, i.e., Mo αCD63 clone H5C6 (Developmental Studies Hybridoma Bank ID AB_528158) and Mo αCD9 (R&D systems #MAB1880), as well as antibodies to detect the neuron-specific enolase (NSE) and albumin, i.e., Rb αNSE clone EPR3377 (Abcam #Ab79757) and Sh αHSA (Abcam #ab8940), were used. HRP-conjugated anti-Mo, anti-Rb, and anti-Sh antibodies were obtained from Jackson ImmunoResearch. Chemiluminescence detection of bands was performed using Pierce ECL Plus Western Blotting Substrate (Thermo Scientific #32132). Finally, the antigens were detected on high-performance films (GE Healthcare #28906844) using the AGFA Curix-60 automatic processor (Agfa, Cologne, Germany).

### RNase protection assay

To detect each miRNA of interest within the EVs, we treated the cleared CSF with proteinase K (Sigma Aldrich #03115879001) or Triton X-100 (TX-100) (Sigma Aldrich #T8787) plus RNase A (Sigma Aldrich # 10109142001), as described in Shelke *et al.* 40, 41. The proteinase K degrades the protein-miRNA complexes and, therefore, facilitates the degradation of the free-circulating miRNAs by the RNase 41. After this treatment, all the miRNAs that are not protected inside the EVs should be degraded. However, the TX-100 permeabilizes the membrane of the EVs and allows the RNase to degrade the miRNAs contained in the vesicles. This assay, in combination with the proteinase K experiment, should show which miRNAs are associated with vesicles or other CSF components. Each procedure was carried out in triplicate. Briefly, a CSF pool of 1.8 mL was divided into nine aliquots of 200 µL. Three of these were incubated with proteinase K (the final concentration, 0.05 mg/mL) at 37 °C for 10 minutes. The reaction was stopped by adding phenylmethylsulfonyl fluoride (the final concentration, 5 mM) (Sigma Aldrich #10837091001) followed by additional heat inactivation (90 °C for 5 minutes). Another three aliquots were treated with 0.1% TX-100. Then, the samples treated with Proteinase K or TX-100 were incubated with RNase A (the final concentration, 0.5 mg/mL) at 37 °C for 20 minutes. The remaining aliquots constituted positive controls; they were not treated and kept at 4 °C until the RNA extraction step. Afterward, total RNA was extracted using NOR, and the levels of the eight miRNAs selected from Yagi *et al.* were determined by RT-qPCR.

### Small RNA sequencing

The quantity and profiles of obtained RNAs were examined using Agilent RNA 6000 Pico Chips (Agilent Technologies #5067-1513). Then, sequencing libraries were prepared using NEXTflex™ Small RNA-Seq Kit v3 (Bioo Scientific Corp. #5132-05) following the protocol for NEXTflex™ Small RNA-Seq Kit v3 V16.06. Briefly, using 70 of 100 µL of each extraction, the total RNA samples were incubated for 2 minutes at 70 °C, then 3′ 4N adenylated adapter and ligase enzyme were added, and ligation was conducted overnight at 20 °C. After removal of excess of 3′-adapter, 5′-adapter was added with ligase enzyme and the mix was incubated at 20°C for 1 hour. The ligation product was used for the reverse transcription with the M-MuLV Reverse Transcriptase in a thermocycler for 30 minutes at 42 °C and 10 minutes at 90 °C. Next, the enrichment of the cDNA was performed using PCR cycling: 2 min at 95 °C; 22-25 cycles of 20 sec at 95 °C, 30 sec at 60 °C, and 15 sec at 72 °C; the final elongation for 2 min at 72 °C and the reaction was stopped at 4 °C. PCR products were resolved on 6% Novex TBE PAGE gels (Thermo Fisher Scientific #EC6265BOX), and the band between 150 bp and 300 bp was cut out from the gel. Small RNAs were extracted from polyacrylamide gel using an adapted protocol, in which the DNA from gel slices was diffused in ddH2O. Afterward, the quantitative and qualitative analyses of the libraries were performed on an Agilent 2100 Bioanalyzer using Agilent High Sensitivity DNA Kit (Agilent Technologies, # 5067-4626) and Qubit dsDNA HS DNA Kit (Thermo Fisher Scientific, # Q32854). The libraries were single-read sequenced for 51 nucleotides in a HiSeq2500 System (Illumina).

### Data analyses

#### Real-Time qPCR assay

The relative quantity of each analyzed miRNA was calculated using the ath-miR-159a spike-in as the reference miRNA and the following formula: ∆Ct=2^ - (Ct miRNA - Ct ath-miR-159a).

#### Small RNA sequencing

FASTQs were trimmed for the adapters following the recommendations of the NEXTflex™ Small RNA-Seq Kit manufacturers. We used Bowtie 42 to align the reads against the human genome with the corresponding annotations (GRCh38/GENCODE-v26), with a mismatch 0, to avoid false positives. GENCODE contains a full set of annotations including all protein-coding *loci* with alternatively transcribed variants, non-coding *loci* with transcript evidence, and pseudogenes 43. Quantification of the transcriptome was performed using Partek expectation maximization, employing Partek Flow application, software version 7.0. Only the miRNAs with 10 or more reads in the three replicas were considered. To estimate the relative miRNA levels from smallRNAseq data, the trimmed mean of M-values normalization method (TMM) was used 44. To visualize the miRNAs identified by each method and their relative abundance, normalized data were represented using the pheatmap package v1.0.10 (default settings) in R 3.4.1 program (2014-04-10, R Foundation for Statistical Computing, Vienna, Austria). UpSet plot showing the total size and overlaps between the miRNAs sets isolated by each method and SEC fractionation was obtained using UpSetR 45 (available online in https:// gehlenborglab.shinyapps.io/upsetr/). We used the smallRNAseq data from Yagi *et al.* as a reference 15. Data were obtained from the NBDC Human Database (dataset ID: JGAS00000000064) (https://humandbs. biosciencedbc.jp/en/) and analyzed in the same way as our data, to be able to compare the two datasets.

#### Correlation analysis

Correlation coefficients between RT-qPCR and smallRNAseq data were calculated using the *cor* function in R 3.4.1 software (2014-04-10, R Foundation for Statistical Computing).

#### Gene target prediction and pathway enrichment analysis

Predicted target genes for each miRNA were obtained using miRWalk 2.0 database 46. The target genes were only selected if they were predicted by at least eight of the twelve miRNA-target prediction programs hosted in the miRWalk 2.0. Pathway enrichment analyses were performed using the over-representation analysis module of the ConsensusPathDB web tool (CPDB) 47. The default collections of Kyoto Encyclopedia of Genes and Genomes (KEGG) 48, Reactome 49, and BioCarta (http:// cgap.nci.nih.gov/Pathways/BioCarta_Pathways) databases were utilized to analyze the predicted target gene lists.

## Results and Discussion

In this study, we compared several existing methods used to enrich the EVs and to analyze their miRNA cargo. We attempted to establish the most suitable technique for detecting CSF miRNAs in 200-µL samples. This volume was chosen considering the realistic amount of CSF that could be obtained from each patient to conduct such studies (200-300 µL).

### Assessing the efficiency of the protocols using Real-Time qPCR

**Figure 2:**
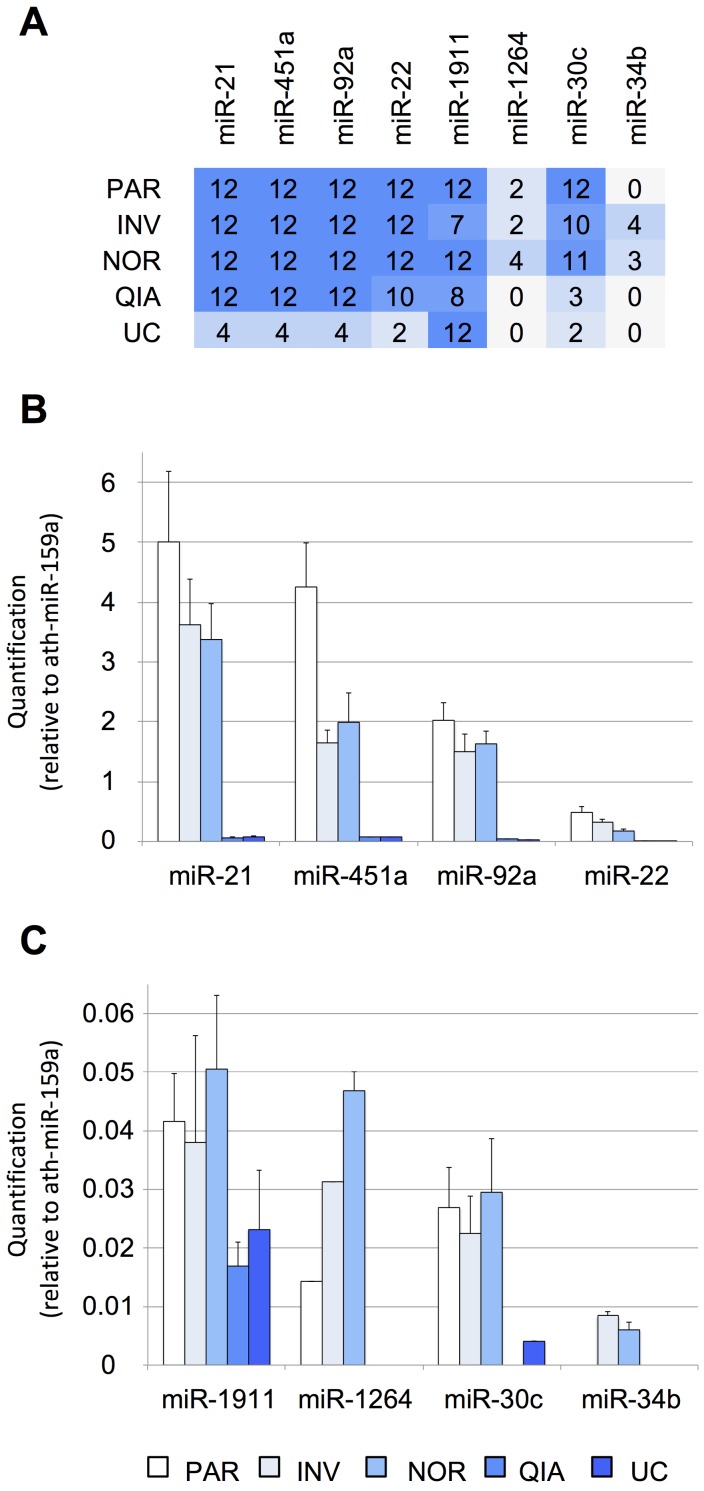
Performance of different protocols measured using RT-qPCR. **A**, the number of TaqMan RT-qPCR replicates (from a total of 12) in which the studied miRNAs were detected. Twelve RT-qPCR reactions were performed for each method (3 EV isolations and miRNA extractions x 2 cDNA synthesis reactions x 2 technical duplicates of each cDNA reaction). **B**, relative quantification (in comparison with ath-miR-159a) of miR-21-5p, miR-451a, miR-92a-3p, and miR-22-3p and **C**, of miR-1911-5p, miR-1264, miR-30c-5p, and miR-34b-3p. Abbreviations: ultracentrifugation (UC), miRCURY Exosome Isolation Kit from Qiagen (QIA), Total Exosome Isolation Reagent from Invitrogen (INV), mirVana PARIS Kit from Ambion (PAR), and Plasma/Serum RNA Purification Kit from Norgen (NOR).

First, the efficiency of the six tested protocols was evaluated using RT-qPCR. Eight miRNAs previously detected by Yagi *et al.* that had variable concentrations in the exosomal fraction (using 7-mL CSF samples) were selected as a reference of the biofluid (Figure S1A), including three EV-associated miRNAs (miR-1911-5p, miR-1264, and miR-34b-3p) and five additional miRNAs identified in both the exosomal and non-exosomal fractions (miR-21-5p, miR-451a, miR-92a-3p, miR-22-3p, and miR-30c-5p) 15. Among these, miR-21-5p, miR-1911-5p, miR-30c- 5p, and miR-34b-3p have also been found in the brain and cerebellum by Meunier *et al.* (GEO accession number: GSE40499) 50. Here, we found that, of the eight evaluated miRNAs in our pool of samples, the four most abundant miRNAs were miR-21-5p, miR-451a, miR-92a-3p, and miR-22-3p. By contrast, miR-1911-5p, miR-1264, miR-34b-3p, and miR-30c-5p, were the least abundant miRNAs in our study (Figure 2). Yagi *et al.* have found the miR-1911-5p in the exosomal fraction and, to a much lesser extent, in the supernatant (Figure S1A). Here, this miRNA was detected by RT-qPCR in the transcriptome obtained by all the protocols, with and without the enrichment step. Similarly, miR-1264, miR-30c-5p, and miR-34b- 3p, detected by Yagi *et al.* only in the exosomal fraction (Figure S1A), were also found using the NOR and INV protocols (Figure 2A). This demonstrated that we could harvest miRNAs of different abundances in the CSF by analyzing as little as 200 µL of fluid. However, these results should be interpreted with caution since Yagi *et al.* have used the CSF from healthy adults 15, not from infants. Additional studies of healthy infants are needed to corroborate our results.

Four of the assessed methods, NOR, INV, PAR, and QIA, detected the more abundant miRNAs in all replicas (12/12), except for miR-22-3p detection by QIA (10/12). The methods using total RNA from the cleared CSF (PAR and NOR) performed slightly better based on our eight reference miRNAs. PAR was the most efficient method for the most abundant miRNAs tested by RT-qPCR (Figure 2B). However, it did not detect the miR-34b-3p, the least abundant miRNA evaluated (Figure S1A). In contrast, NOR was the most effective method when dealing with low copy number, based on the smallRNAseq data of Yagi *et al.* (miR-1911-5p, miR-1264, miR-30c-5p, miR-34b-3p), suggesting superior sensitivity (Figure 2C). Among the methods employing the EV enrichment step, INV obtained the lower Ct values. QIA and UC did not perform well with small CSF samples. However, we cannot rule out that this difference in performance is due to a modification in the INV procedure. INV protocol recommends two consecutive centrifugation steps before adding the exosome isolation reagent (2,000 x *g* for 30 minutes and 10,000 x *g* for 30 minutes). In this study, all the samples were centrifuged at 3,000 x *g* for 15 minutes. Therefore, the INV procedure might have obtained both small (mainly exosomes) and large EVs, while QIA and UC might have achieved more precise exosome separation. This is supported by the fact that the exosomal miR-1911-5p was the most abundant species detected by QIA and UC. In any case, the other methods (NOR, INV, and PAR) performed better in the detection of the reference miRNAs when analyzed by RT-qPCR.

Interestingly, INV and NOR were the only techniques capable of detecting all the miRNAs studied here by RT-qPCR, including CSF miRNAs associated with the vesicular fraction (miR-1911-5p, miR-1264, and miR-34b-3p) (Figure 2A) 15. This suggests that these two are the most proficient methods for the analysis of low copy number. Overall, these results show that each protocol isolates a slightly different group of vesicles and miRNAs.

### Characterization of protein and miRNA subpopulations in CSF using SEC

**Figure 3:**
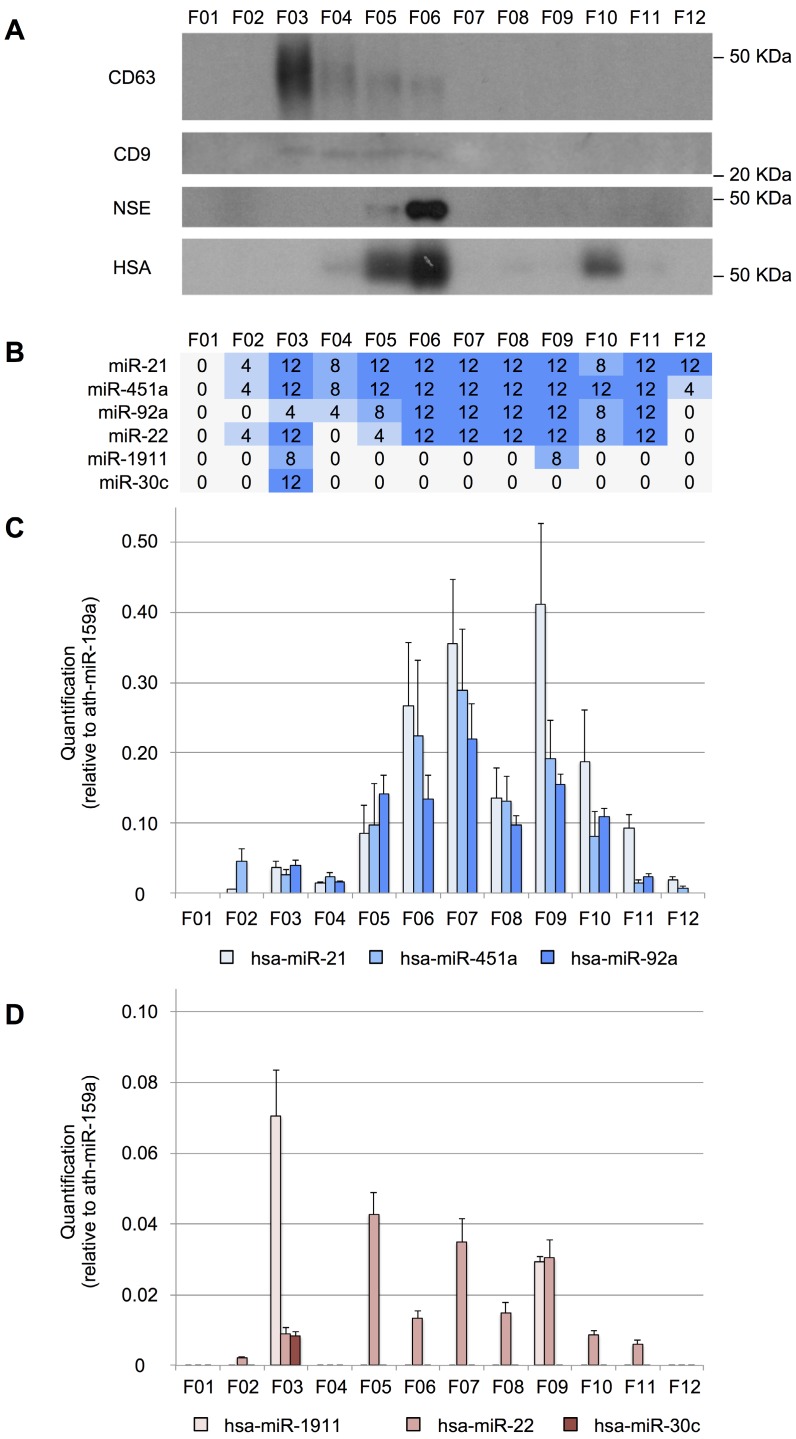
Size-exclusion chromatography analysis of CSF. **A**, Western blot analysis of CD63, CD9, neuron-specific enolase (NSE), and human serum albumin (HSA) proteins. Molecular weights are shown in KDa. **B**, the number of TaqMan RT-qPCR replicates (from a total of 12) in which the studied miRNAs were detected. **C**, quantification (relative to ath-miR-159a) of miR-21-5p, miR-451a, and miR-92a-3p and **D**, of miR-1911-5p, miR-22-3p, and miR-30c-5p.

The miRNAs can have different distributions among the CSF shuttles, such as different populations of EVs, RNA-binding proteins and lipoproteins. To study the distribution of miRNAs among the different CSF structures in infants, we fractionated 200 µL of CSF using an in-house SEC protocol. First, the performance of the method was evaluated by Western blotting (Figure 3A). CD63 and CD9 exosomal markers were predominantly detected in fractions 3 (F03) and fractions 3 to 6 (F03-F06), respectively. These markers were found in earlier fractions than in most of the published reports for SEC 51-53. However, in our SEC method, the sample is separated into fewer fractions than usual, which might explain this discrepancy. The albumin (mostly in F05, F06, and F10) and the NSE (in F05 and mainly in F06) were also detected. Albumin is the most abundant protein in the CSF (245 mg/L) and accounts for 35-80% of the total protein content of this biofluid 54. NSE is a valuable biomarker of brain tumors, used for assessing neuronal damage and formulating the prognosis of brain injury 55. It is upregulated in the biopsies of GBM patients 56. Here, we demonstrated that the level of NSE (found at 8 mg/L in the CSF of healthy individuals 54 could be measured in 200 µL-samples of this fluid. Thus, we showed that our in-house SEC method could be used for fractionating small volumes of CSF.

We performed a RT-qPCR assay to examine the distribution of our eight reference miRNAs among the fractions (Figure 3B). We found that miR-21-5p, miR-451a, and miR-92a-3p were the most abundant in fractions F06, F07, and F09 (Figure 3C). In the case of miR-22-3p, relatively large amounts were obtained in F05, F07, and F09 (Figure 3D). Overall, these miRNAs showed a similar distribution pattern in 12 fractions, where the two main miRNA subpopulations were observed. The first subpopulation, from F05 to F08, co-fractioned with NSE and albumin while the second one (from F09 to F12) co-fractioned with the albumin. Interestingly, the miR-30c-5p was only detected in the fraction F03 (Figure 3D), co-fractionating with the exosomal markers. This is in agreement with the results of Yagi *et al.* who have found that this miRNA is 3 to 5 times more abundant in the vesicular CSF fraction than in the EV-depleted CSF 15. miR-1911-5p, the exosome-associated miRNA, was highly expressed in the exosomal fraction (F03) and to a lesser extent in F09, also in accord with the enrichment in the vesicular fraction reported by Yagi *et al.* 15. By contrast, miR-1264 and miR-34b-3p were not detected using the SEC when analyzing 200-µL CSF samples. These miRNAs were the least abundant references in our study and were detected with fewer than 10 counts by Yagi *et al.* (Figure S1A). Thus, the SEC seems less sensitive than the other methods tested here (PAR, INV, and NOR).

### RNase protection assay

**Figure 4:**
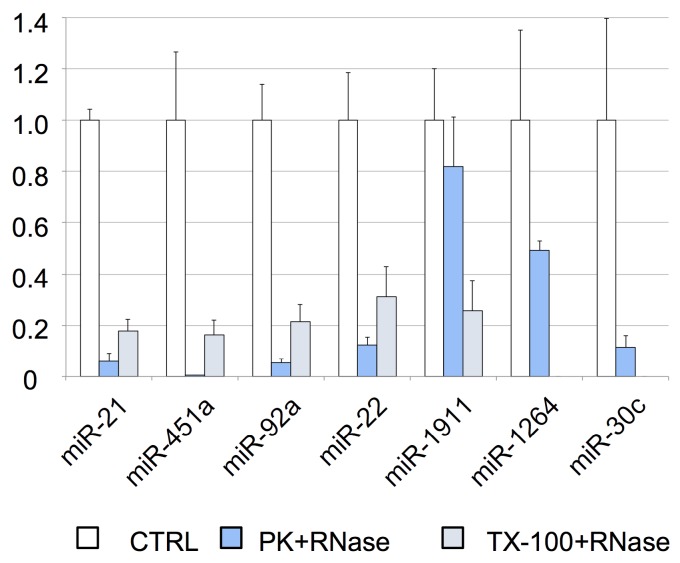
RNase protection assay. Relative quantification (with respect to ath-miR-159a) of each miRNA evaluated by RT-qPCR. The positive control (CTRL) shows the total abundance of each miRNA in this pool of samples. The samples were also treated with proteinase K and RNase A (PK+RNase) or Triton X-100 and RNase A (TX-100+RNase) to examine the association of miRNAs with the vesicles (and their location inside the vesicles or on their surface). The three conditions were assessed in triplicate.

To further analyze the association of miRNAs with vesicles and their exact location (inside or on the surface of the vesicles), we performed an RNase protection assay. The cleared CSF was treated with Proteinase K or TX-100, and RNase A. We found that most of the miRNAs studied by RT-qPCR (i.e., miR-21-5p, miR-451a, miR-92a-3p, and miR-22-3p) were degraded after proteinase K and RNase A treatment (Figure 4). Therefore, their levels should be higher in the supernatant than inside the EVs. Although the miR-30c was degraded in the absence of TX-100 (Figure 4), this miRNA was associated with the vesicular fraction containing CD63 (F03) in our SEC experiment (Figures 3A and 3D). The degradation observed after the treatment with proteinase K might be explained by the association of this miRNA with the outer surface of the vesicles. The treatment with TX-100 permeabilizes the membranes, allowing the RNase degradation of miRNAs inside the EVs. It resulted in complete degradation of miR-1264 and of almost all the miR-1911-5p. By contrast, after proteinase K treatment no more than half of these miRNAs were degraded, suggesting that a large proportion of miR-1264 and miR-1911-5p is protected within EVs. This agrees with the results of Yagi *et al.* 15 and our miR-1911-5p data (Figure 4). In summary, our results show that miRNAs in CSF are both free-floating and associated with EVs, on the surface of these vesicles or inside them.

### CSF miRNA profiling using smallRNAseq

The methods identifying the majority of the eight reference miRNAs, using RT-qPCR (Figure 2), were further analyzed. We employed smallRNAseq to establish which of these techniques could detect the largest number of miRNAs (NOR, INV, or PAR). The fractions F03 and the F09 of the SEC were also sequenced. We selected the F03 since it contained the largest CD63 exosome population as shown by the Western blot analysis (Figure 3A), to identify miRNAs enriched in the exosomal fraction. F09 was also sequenced as a non-vesicular fraction for the comparison with F03. As expected, our results showed that different protocols yielded a different number of reads (Table S1).

The smallRNAseq data normalization remains a hot topic that still needs to be addressed. Several approaches have been evaluated. For example, adding a synthetic oligonucleotide during RNA extraction might serve as an indicator of technical variability. Here we spiked with the cel-miR-39 after CSF denaturing process; however, the amounts of the cel-miR-39 oligonucleotide recovered after smallRNAseq were highly variable (in raw reads, NOR= 1,421-2,914; INV= 1,450-3,083; PAR= 676-933; F03= 285,077-1,502,484; and F09= 249-647). Another normalization approach employs non-variable small RNAs as endogenous controls. To date, several such miRNAs have been proposed to normalize data in RT-qPCR experiments involving CSF (let-7c, miR-21, miR-24, miR-99b, miR-125, miR-328, miR-1274, RNU6B, and RNU44) 8. Sorensen *et al.* found the best normalizer as the average Ct value of the 9 miRNAs detected in all samples 31. However, none of the small RNAs has shown stable levels in all pathological conditions and CSF components 19. Sorensen *et al.* used the trimmed mean of M-values normalization (TMM) for NGS data normalization 31, 44. Yagi *et al.* have presented their data in counts per million after applying a normalization factor based on the relative log expression method 15. In other words, there is no standard normalization strategy 20. Establishing a common approach is crucial if we are to make unbiased comparisons between studies 8.

**Figure 5:**
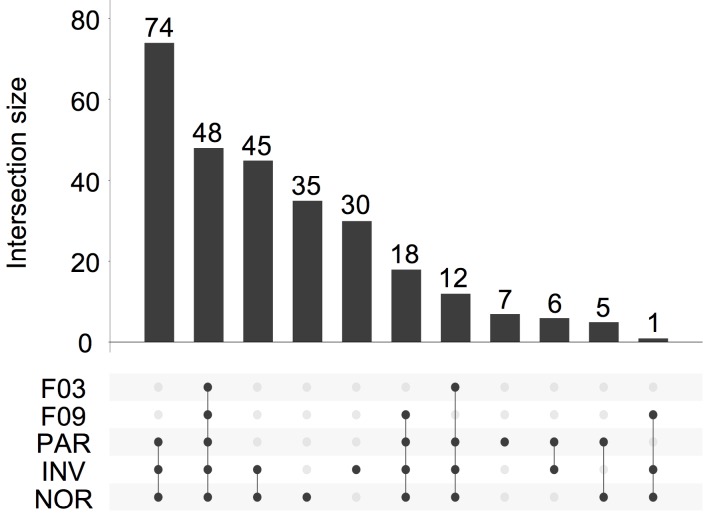
UpSet plot showing the total set size and overlaps between the 281 unique miRNAs and those isolated by each method (PAR, INV, and NOR) or found in the F03 and F09 fractions of the SEC. The number of common miRNAs detected by each method is indicated on the y-axis. The shaded circles connected by solid lines in the lower panel show the intersecting miRNA datasets.

**Figure 6:**
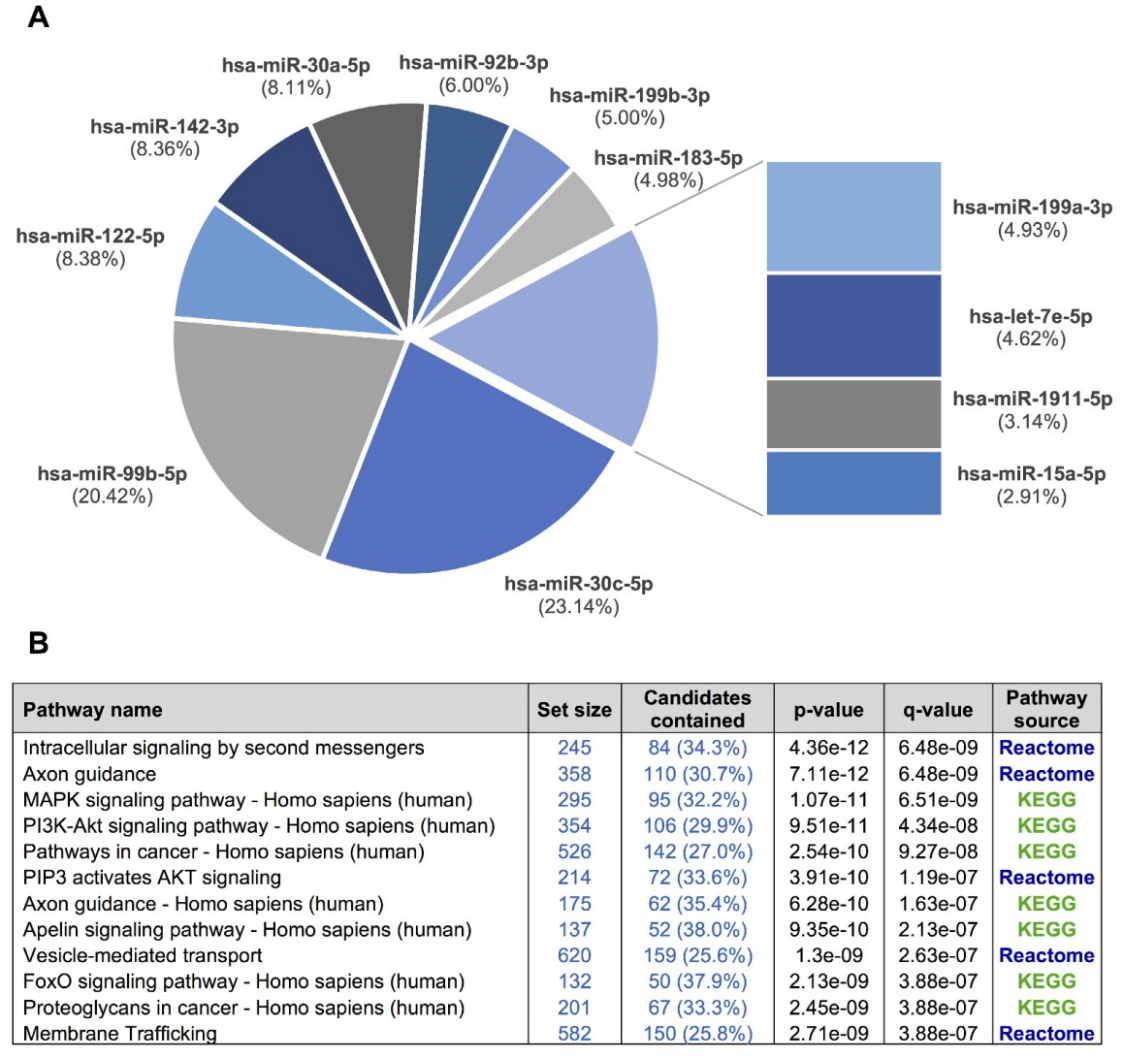
**A**, a pie chart showing the 12 miRNAs detected in the exosomal fraction but not in the F09 fraction of the size-exclusion chromatography. **B**, the most representative pathways predicted by ConsensusPathDB considering the 3,638 target genes of these 12 miRNAs.

Overall, 281 different miRNAs were identified (the sum of all unique miRNAs detected in our study), and the FASTQ data were made available in GEO (accession number GSE122068). Our comparisons showed that NOR and INV methods identified the largest number of miRNAs (238 and 234, respecttively). Of these, 198 were detected by both methods. However, NOR identified a subpopulation of miRNAs that were not detectable by INV and vice versa (35 and 30 miRNAs, respectively) (Figure 5 and Figure S2). Interestingly, one of the EV-enriched methods, the INV, revealed a 30‑miRNAs subpopulation not detectable by the other methods (Table S2). PAR identified 170 miRNAs, and only 7 of them were exclusively detected by this method. In the SEC fractions F03 and F09, 60 and 67 miRNAs were detected, respectively (Figure 5). Of these, 48 miRNAs were common for the two fractions (Figure 5 and Table S3). However, we identified a subset of 12 miRNAs in the vesicular fraction (F03) not found in the supernatant (F09) that could be considered vesicle-associated miRNAs (Figure 6).

It is remarkable that all the miRNAs detected in the SEC fractions were also found using the NOR and INV protocols (Figure S2). We can conclude that both the NOR and INV protocols are suitable for analyzing large catalogs of vesicular and non-vesicular miRNAs in small CSF samples from infants.

We compared our smallRNAseq data with the data of Yagi *et al.* (Figure S1B). We identified 86 out of the 92 miRNAs found by these authors after applying our cut-off criterion (10 or more counts) (Figures S1B and S1C). For the detection of exosomal miRNAs, NOR and INV were the methods that best harvested the Yagi's exosome-associated miRNAs from only 200 µL of fluid (78/85 and 77/85, respectively). However, we failed to detect several miRNAs found by Yagi (NOR = 7/85 and INV = 8/85) (Figure S1C). These miRNAs were detected at low levels in the study of Yagi *et al.* even though they used 7-mL samples of the fluid (all of them obtained less than 65 raw reads). Their low abundance is the most likely reason for missing these molecules in small-volume samples. By contrast, we identified a large number of miRNAs that Yagi *et al.* have not detected, using NOR and INV (160 and 157, respectively). This suggests that these protocols perform better than the UC method used by Yagi. However, our results should be interpreted with caution since those authors have analyzed samples from healthy adults (aged 37-79) rather than from infants. Therefore, the discrepancies between the two sets of data might be due to biological variability among subjects, apart from the differences between the protocols. Thus, further studies using samples from healthy infants are needed to confirm our results.

### Reproducibility assessment

To test the reproducibility of the RT-qPCR assays, the coefficient of variation (CV, %) was calculated for each of the eight reference miRNA and method (standard deviation/mean of quantification, relative to ath-miR-159a)*100. Only the miRNAs detected in all the replicas were considered. NOR had the lowest coefficient of variation (25.6%), followed by INV and PAR (33.0 and 40.5%, respectively). The results for spiked-in cel-miR-39 showed the variability of 15.3% when using NOR. By contrast, in this case, INV showed the highest variability (69.5%) and PAR method was not considered since it only detected this miRNA in 6 out of 10 replicas (Table 1).

**Figure 7:**
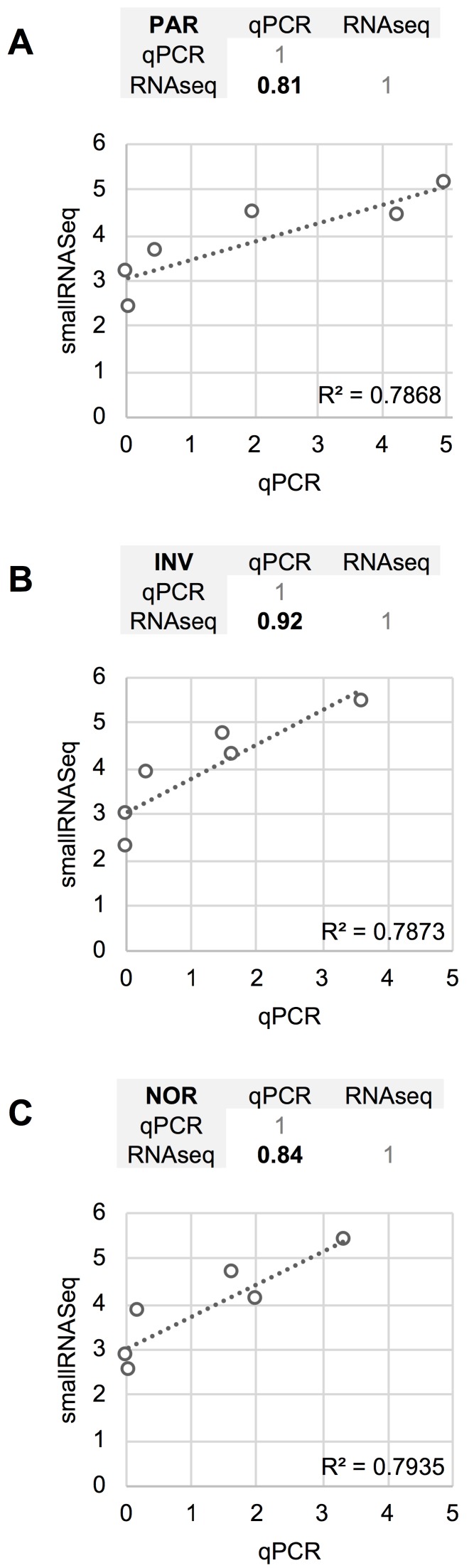
Comparison between the quantification (relative to ath-miR-159a) obtained using RT-qPCR and the normalized smallRNAseq counts (represented on a logarithmic scale) for six reference miRNAs considered and method compared. **A**, mirVana PARIS Kit from Ambion. **B**, Total Exosome Isolation Reagent from Invitrogen. **C**, Plasma/Serum RNA Purification Kit from Norgen. The miR-1264 and miR-34b-3p were not considered. The correlation coefficients between RT-qPCR and smallRNAseq data for each method are also shown (upper panel for each method).

The differences between RT-qPCR and smallRNAseq data were also examined. Although most of the miRNAs found using RT-qPCR were also identified by smallRNAseq, miR‑34b-3p was detected by INV and NOR only in 2/3 of the replicates. Similarly, in F03, miR-1264 was found in only 2/3 of the replicates. After applying our detection criterion (10 or more counts), miR-1264 and miR-34b-3p were treated as non-detected. However, these two miRNAs were identified by RT-qPCR when the samples were processed using PAR, INV, and NOR protocols (Figure 2). This confirmed the opinion that RT-qPCR is suitable for low copy number RNA samples and samples with undefined normalizer molecules, such as the CSF 8. The correlation coefficients for the six miRNAs were 0.81 for PAR, 0.84 for NOR, and 0.92 for INV, showing good agreement between both strategies (Figure 7). Our results are in accord with the report of Yagi *et al.* who also evaluated the consistency of these techniques 15.

### Gene target prediction and pathway enrichment analysis

We used our smallRNAseq data to predict the target genes and the related pathways in which they might be involved. We identified 9,952 target genes for the 281 miRNAs detected in our CSF study by smallRNAseq, which were then used to perform pathway enrichment analyses employing the default collections of KEGG, Reactome, and BioCarta. The three most significant pathways were axon guidance (p = 2.83e-23), membrane trafficking (p = 5.05e-22), and vesicle-mediated transport (p = 7.36e-21). A complete list of predicted pathways is shown in Table S4. The membrane trafficking and vesicle-mediated transport pathways are involved in the release and internalization of various components from the extracellular space by the EVs. The axon guidance pathway was also overrepresented. The axon guidance process is an important event in neurodevelopment; it is related to neuron maturation and the formation of neuronal connections in the first years of life 57. We repeated this analysis using the 3,638 target genes of the 12 miRNAs detected in the exosomal fraction (F03) but not in the F09 (Figure 6). Overall, the identified pathways are consistent with the source of samples for our study, in which the EV-associated miRNAs were analyzed in a cohort of infants from 0 to 7 years old. Our results indicate that the analysis of miRNAs in CSF fluid might become a useful, minimally invasive tool to examine the physiological and pathological processes affecting brain performance.

### NOR as a potential tool in CSF diagnosis

Overall, the NOR and INV methods obtained the best results in the two analyses performed (RT-qPCR and smallRNAseq). The main difference between NOR and INV is the miRNA isolation procedure. Two important aims of standardization should be the simplicity and reproducibility of the protocol. Among the methods tested here, NOR used the easiest and shortest protocol (as can be seen in Figure S3). It also was the most reproducible method when tested with RT-qPCR, and showed a good correlation between RT-qPCR and smallRNAseq techniques. Moreover, this protocol detected more miRNAs than the INV. Therefore, this might be the best method to analyze small-volume samples of CSF.

## Figures and Tables

**Table 1:** Coefficients of variation (CV, %) for each miRNA and method evaluated.

miRNA	PAR			INV			NOR	
	N	CV (%)		N	CV (%)		N	CV (%)
cel-miR-39				10	69.5		10	15.3
hsa-miR-21-5p	10	39.1		10	22.2		10	30.5
hsa-miR-451a	10	35.4		10	24.0		10	24.2
hsa-miR-92a-3p	10	36.6		10	27.4		10	30.8
hsa-miR-22-3p	10	52.7		10	21.9		10	13.9
hsa-miR-1911-5p								
hsa-miR-1264								
hsa-miR-30c-5p	10	38.7					10	39.1
hsa-miR-34b-3p								
Mean CV (%)	40.5 ± 7.0			33.0 ± 20.5			25.6 ± 9.8	

NOR had the lowest coefficient of variation, followed by the INV and PAR methods. Only the cases in which the miRNAs were detected in all 10 replicates were considered. Abbreviations: number of replicates analyzed (N), mirVana PARIS Kit from Ambion (PAR), Total Exosome Isolation Reagent from Invitrogen (INV), and Plasma/Serum RNA Purification Kit from Norgen (NOR).

## Abbreviations

CSF: cerebrospinal fluid
CPDB: ConsensusPathDB web tool
EVs: extracellular vesicles
F01-F12: fractions 1 to 12 of the size-exclusion chromatography
GBM: glioblastoma
GEO: Gene Expression Omnibus
INV: Total exosome isolation reagent
KEGG: Kyoto Encyclopedia of Genes and Genomes
miRNA: microRNA
NOR: Plasma/Serum RNA Purification Kit
NSE: neuron-specific enolase
PAR: mirVana PARIS Kit
QIA: miRCURY Exosome Isolation Kit - Cells, Urine and CSF
RT-qPCR: real-time PCR
SEC: size-exclusion chromatography
smallRNAseq: small RNA sequencing
TX-100: Triton X-100
UC: ultracentrifugation
