# Supplementary material for: A Comprehensive Study of Vesicular and Non-Vesicular miRNAs from a Volume of Cerebrospinal Fluid Compatible with Clinical Practice
Source: Theranostics. 2019 Jun 19;9(16):4567–79. doi: 10.7150/thno.31502 (PMC6643433; doi:10.7150/thno.31502)
Supplement: Supplementary file 1 — Supplementary figures and tables. [file thnov09p4567s1.pdf]

SUPPLEMENTARY MATERIALS

A

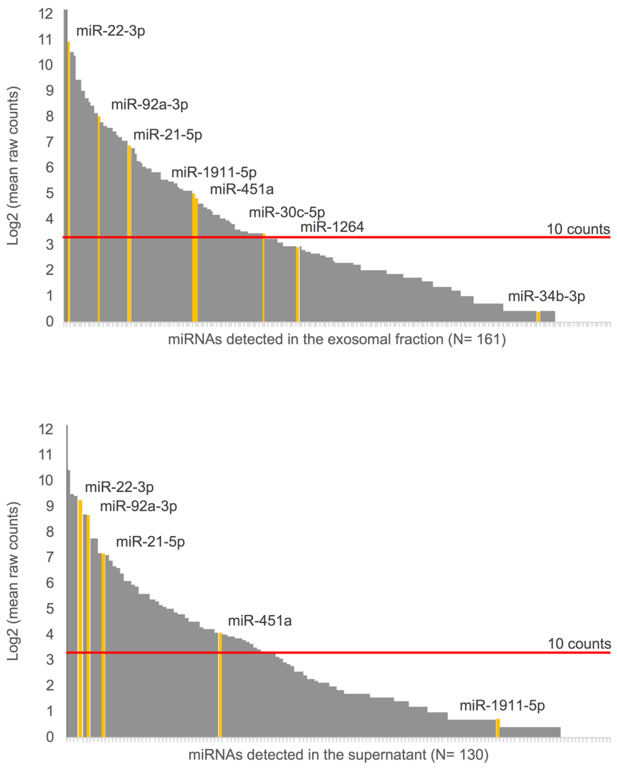

B

Study	N	Age	Volume	Method	miRNAs (≥10 counts)	Number of unique miRNAs
Yagi et al.	3	37-79	7 mL	UC	85 (Exo.)<br>65	92
Present study	7	0-7	200 µL	NOR<br>INV<br>PAR<br>SEC-F09<br>SEC-F03	238<br>234<br>170<br>67<br>60	281

C

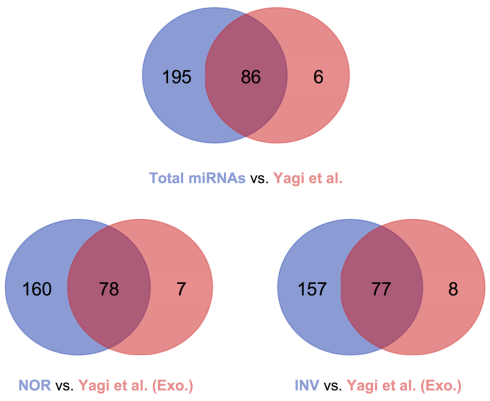

Figure S1. Comparison of the data obtained here with the results of Yagi et al. [15]. **A**, each miRNA identified detected by Yagi et al. (x-axis) and the number of raw reads on a logarithmic scale (y-axis). Only the eight miRNAs selected as reference miRNAs for this study are highlighted in orange. The horizontal line indicates the threshold chosen to accept a miRNA as detected (10 or more counts). **B**, the summary of the two studies and the number of miRNAs detected by each method. **C**, Venn diagrams showing the number of common miRNAs detected between our study and the one by Yagi et al. in the exosomal fraction (Exo.) (*upper part*), as well as by the NOR and INV protocols in comparison with Yagi et al. (*lower part*). Abbreviations: ultracentrifugation (UC), miRCURY Exosome Isolation Kit from Qiagen (QIA), Total Exosome Isolation Reagent from Invitrogen (INV), mirVana PARIS Kit from Ambion (PAR), and Plasma/Serum RNA Purification Kit from Norgen (NOR).

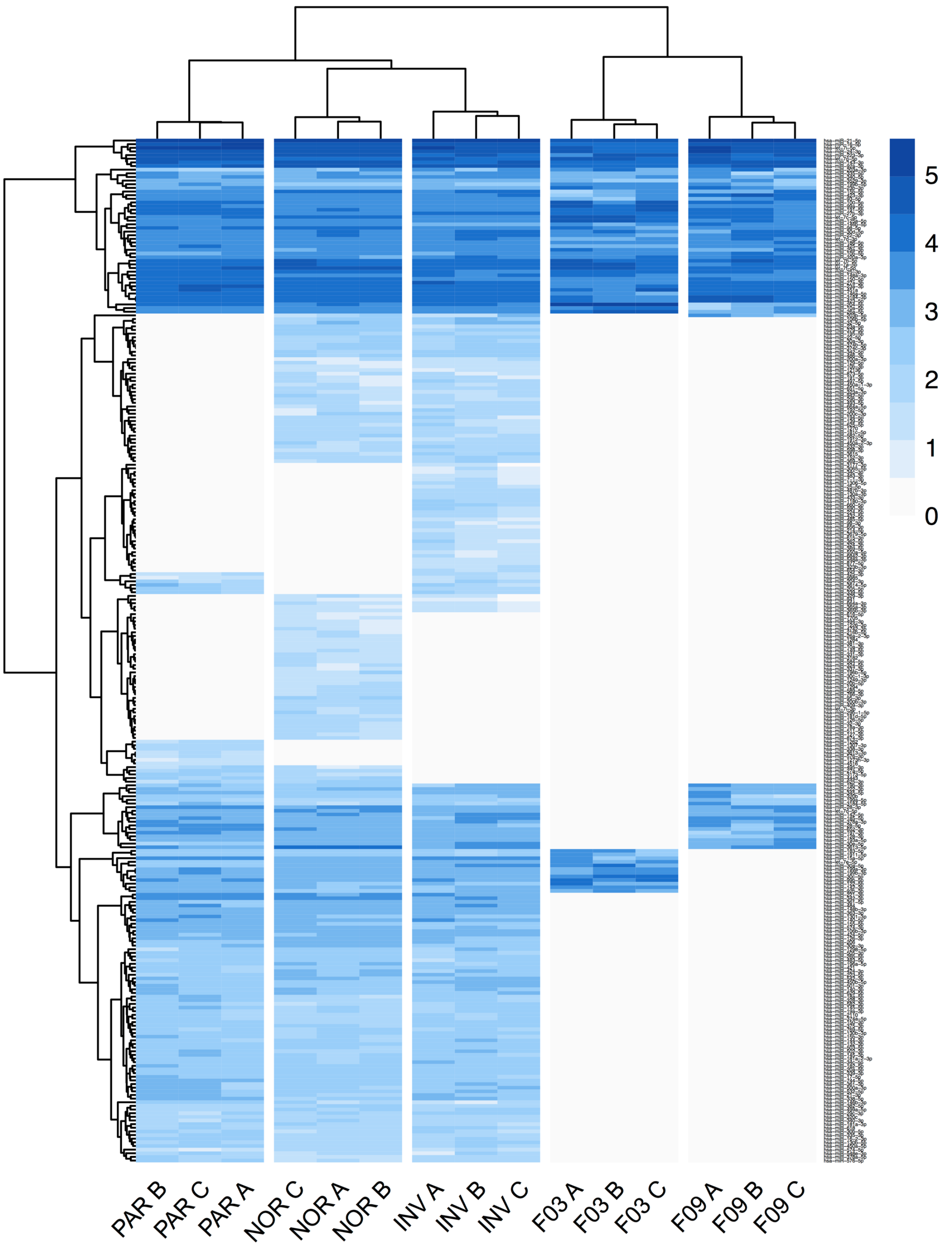

Figure S2. Heatmap representing the abundance of each miRNA for the compared methods. The smallRNAseq counts (10 or more) were TMM-normalized and represented on the logarithmic scale. The darkest blue bars represent the highest abundance and the white bars, the lowest. Each method was assessed in triplicate.

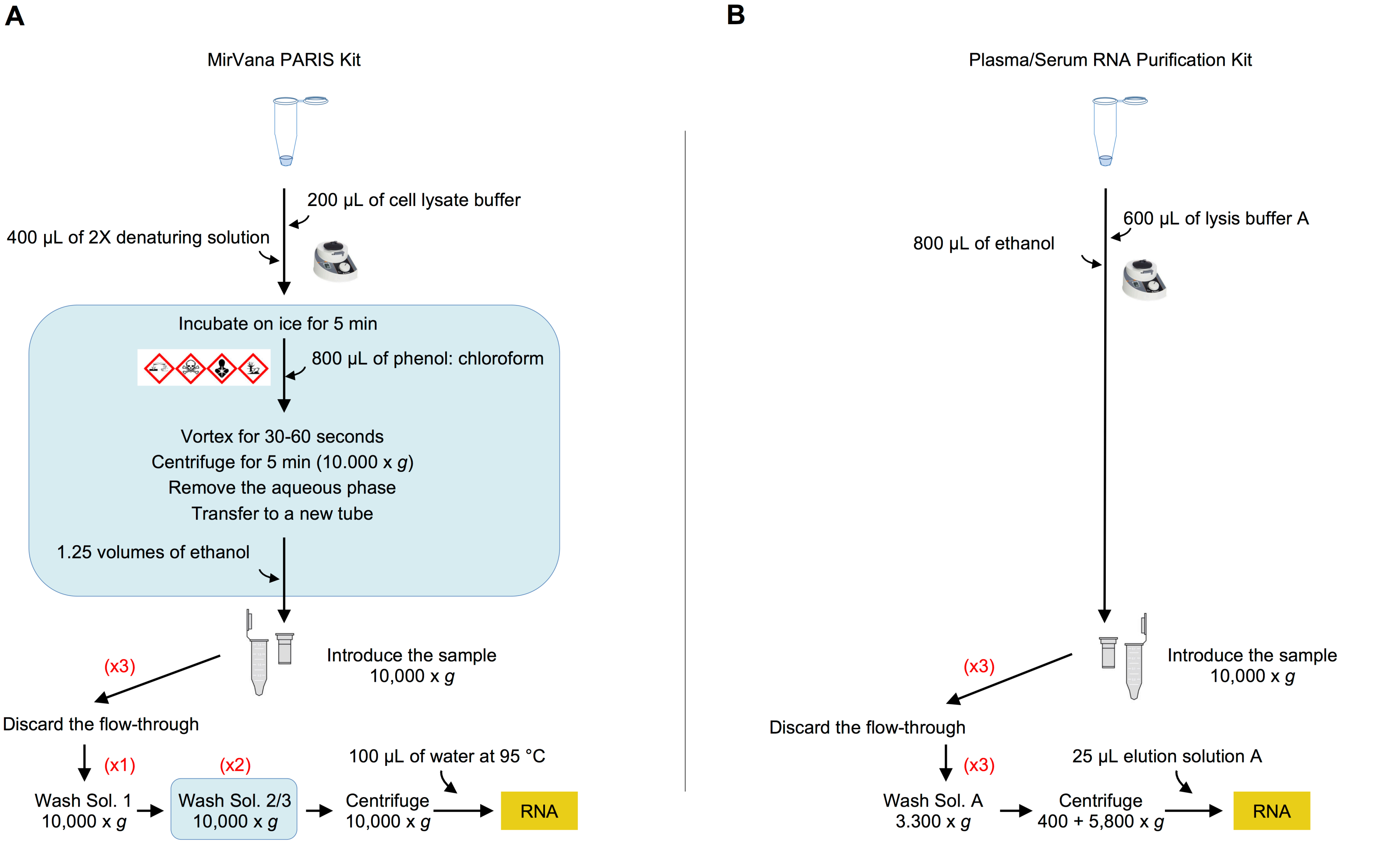

Figure S3. Comparison of the two RNA extraction protocols used in this work: **A**, using mirVana PARIS Kit from Ambion and **B**, using the Plasma/Serum RNA Purification Kit from Norgen.

Table S1. Results of sequencing and computational post-processing. The green color represents the highest percentage of alignment against the human genome and the red one, the lowest. Abbreviations: mirVana PARIS Kit from Ambion (PAR), Total Exosome Isolation Reagent from Invitrogen (INV), Plasma/Serum RNA Purification Kit from Norgen (NOR), and the fractions 3 (F03) and 9 (F09) of the SEC.

Method	Sample	Total reads	Reads aligned with the genome		Read counts aligned with miRBase22		Average coverage depth	Average length	Average quality	%GC
			N	%	N	%				
DIR	DIR_A	26438319	18470770	69.9	1249070	0.1	104.4	25.8	37.0	52.7
	DIR_B	28440884	18818832	66.2	1392689	0.2	74.7	24.5	37.0	53.0
	DIR_C	30495052	19466510	63.8	1086513	0.2	74.5	23.7	37.0	53.6
F03	F03_A	25958614	9004682	34.7	243519	0.1	60.5	20.2	36.8	55.2
	F03_B	19540619	9312030	47.7	65634	0.2	25.0	18.2	36.8	55.4
	F03_C	25243946	10856518	43.0	84024	0.4	17.2	18.4	36.8	55.7
F09	F09_A	22831445	10171399	44.5	140001	0.2	36.8	18.5	36.8	56.3
	F09_B	24304724	11901408	49.0	121530	0.2	34.4	18.3	36.8	56.0
	F09_C	21646255	10867442	50.2	938047	0.1	87.2	23.1	37.0	53.2
INV	INV_A	27875522	14743858	52.9	1966654	0.3	33.6	18.8	36.8	53.1
	INV_B	22467111	16583184	73.8	6942463	0.2	68.5	21.0	36.9	48.5
	INV_C	26656900	18972809	71.2	7027026	0.2	74.9	20.9	36.9	49.4
NOR	NOR_A	32689429	29545751	90.4	1890229	0.1	267.9	29.5	37.2	50.9
	NOR_B	32771511	25542919	77.9	1511752	0.3	77.9	27.6	37.1	51.1
	NOR_C	34307902	28037677	81.7	1306748	0.2	119.1	28.1	37.1	51.5

Table S2. The complete list of the 234 miRNAs detected by smallRNAseq using the INV protocol and their TMM-normalized counts obtained using the INV protocol. The 30 miRNAs that were specifically detected by INV are highlighted in blue and marked with an asterisk (*). INV_A, INV_B, and INV_C indicate each of the triplicates analyzed.

Name	Normalized counts		
	INV_A	INV_B	INV_C
*hsa-miR-1180-3p	253.22	134.10	57.92
*hsa-miR-660-5p	163.73	174.58	77.13
*hsa-miR-590-3p	206.95	116.67	27.61
*hsa-miR-433-3p	68.14	157.00	97.34
*hsa-miR-130a-3p	82.37	143.61	50.09
*hsa-miR-487b-3p	81.86	60.64	117.12
*hsa-miR-362-5p	197.80	29.67	11.10
*hsa-miR-432-5p	114.92	88.59	30.88
*hsa-miR-224-5p	106.78	53.30	32.87
*hsa-miR-324-3p	135.26	15.12	26.90
*hsa-miR-3065-5p	5.08	150.67	11.67
*hsa-miR-1306-5p	65.09	80.52	11.53
*hsa-miR-25-5p	44.24	78.36	25.33
*hsa-miR-7-1-3p	24.41	111.20	10.67
*hsa-miR-654-3p	60.51	16.85	65.89
*hsa-miR-369-5p	81.86	23.48	25.47
*hsa-miR-323b-3p	20.85	21.17	80.40
*hsa-miR-656-3p	77.80	23.77	7.97
*hsa-miR-3679-5p	58.98	36.44	13.80
*hsa-miR-615-3p	55.42	33.56	13.23
*hsa-miR-6842-3p	63.56	3.60	25.62
*hsa-miR-98-3p	55.93	12.24	20.21
*hsa-miR-214-5p	38.64	31.40	13.52
*hsa-miR-548a-3p	29.49	13.68	33.02
*hsa-miR-877-5p	20.85	27.08	26.18
*hsa-miR-483-3p	21.86	40.62	10.67
*hsa-miR-485-5p	39.15	13.68	9.82
*hsa-miR-34c-3p	12.71	38.46	10.39
*hsa-miR-3177-3p	28.98	23.91	1.57
*hsa-miR-550a-5p	19.32	5.91	27.04
hsa-miR-21-5p	199674.68	305318.16	334780.32
hsa-miR-16-5p	83888.17	61814.95	57789.17
hsa-let-7i-5p	124088.43	35838.43	42205.76
hsa-miR-92a-3p	48643.03	61627.41	51851.52
hsa-miR-24-3p	47785.73	38130.85	60769.24
hsa-miR-143-3p	25078.64	25016.34	34675.98
hsa-miR-27a-3p	35869.55	24684.32	14426.16
hsa-miR-223-3p	19892.16	31949.18	21076.48
hsa-let-7g-5p	39505.17	14256.32	17936.75
hsa-miR-3184-3p	21067.25	16845.03	21471.67
hsa-miR-423-5p	21067.25	16845.03	21471.67
hsa-miR-451a	22932.35	23778.74	12567.76
hsa-let-7f-5p	23255.74	13059.05	21055.85
hsa-let-7b-5p	14439.75	21115.56	21195.17

hsa-miR-23a-3p	20689.96	14972.35	12344.62
hsa-let-7a-5p	11938.04	8124.06	13613.01
hsa-miR-146a-5p	10510.75	12164.13	9982.60
hsa-miR-27b-3p	14672.13	8085.89	8891.81
hsa-miR-204-5p	15340.27	7435.40	7277.19
hsa-miR-30d-5p	5132.58	12326.17	8564.36
hsa-miR-25-3p	13450.26	6155.60	6129.90
hsa-miR-148a-3p	8354.29	6959.49	9244.74
hsa-let-7d-3p	6515.13	9815.54	6892.39
hsa-miR-22-3p	3580.19	8643.04	9894.80
hsa-miR-15b-5p	4621.05	8649.09	8116.81
hsa-miR-423-3p	4454.77	6535.43	8143.27
hsa-miR-191-5p	5827.66	7074.15	5338.96
hsa-miR-29a-3p	5831.22	7083.94	4605.65
hsa-miR-100-5p	6487.67	5880.48	4422.92
hsa-miR-425-5p	4725.28	6760.71	5268.37
hsa-let-7c-5p	5298.85	4772.95	6529.22
hsa-miR-320a-3p	4065.28	8233.25	4122.09
hsa-miR-186-5p	4016.47	7281.85	4393.47
hsa-miR-99a-5p	4990.71	6309.86	3647.63
hsa-miR-93-5p	2654.76	6728.16	5495.64
hsa-miR-340-5p	3294.94	7175.55	3898.24
hsa-miR-3613-5p	1418.14	6751.49	5825.51
hsa-miR-10a-5p	2619.68	5961.57	4675.09
hsa-miR-26a-5p	2845.95	4359.26	4602.80
hsa-miR-125a-5p	3562.90	4977.20	3131.77
hsa-miR-150-5p	3398.67	3884.07	2614.62
hsa-miR-15a-5p	4436.47	2724.11	2389.35
hsa-miR-98-5p	4871.22	2223.56	2362.17
hsa-miR-30e-5p	1246.79	4665.49	3454.80
hsa-miR-10b-5p	1773.57	4260.16	3088.50
hsa-miR-335-5p	1936.79	3350.54	2689.19
hsa-miR-142-5p	1146.11	3511.87	2426.63
hsa-miR-221-3p	2740.70	1723.60	1717.80
hsa-miR-30a-5p	730.68	3307.47	2118.68
hsa-miR-342-3p	1601.20	2396.12	1799.91
hsa-miR-203a-3p	2190.52	527.62	3000.13
hsa-miR-28-3p	1717.13	2031.27	1901.66
hsa-miR-378a-3p	1361.70	2355.65	1633.27
hsa-miR-146b-5p	1647.98	1741.31	1837.33
hsa-miR-484	1101.87	2338.51	1673.11
hsa-miR-185-5p	558.31	2370.77	2111.28
hsa-miR-652-3p	1598.15	1436.23	1524.12
hsa-let-7d-5p	1202.55	1982.44	1369.57
hsa-miR-361-5p	892.89	2061.95	1368.15
hsa-miR-140-5p	2619.17	742.24	742.85
hsa-miR-99b-5p	2129.00	1199.00	714.95
hsa-miR-199a-3p	1661.71	1523.09	806.46
hsa-miR-199b-3p	1654.59	1490.83	812.01
hsa-miR-148b-3p	1471.03	1081.75	1351.92
hsa-miR-363-3p	999.16	1423.41	1155.11
hsa-miR-128-3p	1155.77	1246.24	1146.72
hsa-miR-23b-3p	1624.59	929.50	932.97

hsa-miR-424-5p	894.41	1425.86	921.58
hsa-miR-197-3p	1001.70	1453.95	698.30
hsa-miR-106b-3p	865.94	1377.03	903.51
hsa-miR-450b-5p	652.38	1543.11	949.48
hsa-miR-193a-5p	980.35	1331.66	753.66
hsa-miR-3529-3p	1644.42	718.19	653.48
hsa-miR-30c-5p	1249.33	959.17	733.74
hsa-miR-501-3p	504.92	1320.28	951.04
hsa-miR-28-5p	1299.16	845.52	562.12
hsa-miR-542-3p	496.27	1073.83	993.59
hsa-miR-1301-3p	1697.30	538.86	220.72
hsa-miR-222-3p	752.04	890.89	698.45
hsa-miR-345-5p	743.39	759.82	795.07
hsa-let-7e-5p	698.14	658.41	742.70
hsa-miR-142-3p	520.68	801.01	738.29
hsa-miR-122-5p	1298.14	254.23	505.19
hsa-miR-21-3p	895.94	505.01	553.01
hsa-miR-206	820.17	571.12	556.28
hsa-miR-338-5p	791.19	609.44	520.85
hsa-miR-126-3p	811.53	550.09	536.93
hsa-miR-92b-3p	481.53	664.89	675.25
hsa-miR-361-3p	375.26	871.59	506.47
hsa-miR-19b-3p	132.20	831.69	780.13
hsa-miR-574-3p	305.60	879.66	524.69
hsa-miR-32-5p	280.68	664.61	751.10
hsa-miR-320b	432.21	356.07	785.25
hsa-miR-223-5p	723.06	443.50	395.47
hsa-miR-30e-3p	567.46	441.77	528.25
hsa-miR-199b-5p	806.45	400.72	292.44
hsa-miR-34c-5p	726.61	532.95	234.81
hsa-miR-106b-5p	252.21	667.77	515.72
hsa-miR-144-3p	502.38	620.53	255.30
hsa-miR-500a-3p	227.29	525.17	601.25
hsa-miR-629-5p	357.97	361.98	629.28
hsa-miR-421	418.99	390.21	528.82
hsa-miR-1298-5p	136.27	796.69	394.19
hsa-miR-424-3p	147.46	591.86	529.24
hsa-miR-502-3p	259.32	594.17	325.88
hsa-miR-532-5p	312.71	581.35	278.50
hsa-miR-140-3p	187.12	603.53	320.62
hsa-miR-181a-2-3p	492.72	357.51	219.58
hsa-miR-486-3p	563.39	293.70	187.42
hsa-miR-203b-5p	15.25	13.54	1003.41
hsa-miR-155-5p	348.31	366.87	256.87
hsa-miR-3184-5p	555.26	155.28	222.00
hsa-miR-483-5p	38.64	483.11	377.54
hsa-miR-199a-5p	514.07	222.69	162.09
hsa-miR-182-5p	402.21	169.39	311.23
hsa-miR-150-3p	230.34	193.74	416.82
hsa-miR-339-3p	270.51	211.02	356.77
hsa-miR-145-3p	451.53	236.80	138.61
hsa-miR-20a-5p	326.44	205.98	287.03
hsa-miR-29c-3p	238.98	284.19	266.83

hsa-miR-17-5p	399.16	280.88	95.20
hsa-miR-26b-5p	60.00	363.27	349.51
hsa-miR-769-5p	148.48	467.70	155.54
hsa-miR-582-3p	126.10	437.74	207.06
hsa-miR-499a-5p	156.61	256.83	348.80
hsa-miR-132-3p	269.49	261.72	226.98
hsa-miR-503-5p	175.42	340.95	226.41
hsa-miR-328-3p	186.61	373.21	147.43
hsa-miR-15b-3p	372.21	163.05	131.21
hsa-miR-145-5p	68.14	342.24	254.30
hsa-miR-744-5p	227.80	252.22	184.43
hsa-miR-196a-5p	170.85	116.53	373.70
hsa-miR-375-3p	240.51	200.65	202.50
hsa-miR-195-5p	489.16	50.27	54.08
hsa-miR-130b-3p	228.82	192.01	165.93
hsa-miR-342-5p	208.48	167.95	191.55
hsa-miR-339-5p	335.60	149.37	81.54
hsa-miR-3615	175.93	158.45	217.02
hsa-miR-374b-5p	160.17	218.94	168.78
hsa-miR-374c-3p	160.17	218.94	168.78
hsa-miR-183-5p	228.31	150.67	165.22
hsa-miR-1911-5p	167.29	159.02	191.12
hsa-miR-19a-3p	54.92	244.15	216.31
hsa-miR-505-3p	87.46	326.11	85.53
hsa-miR-18a-5p	213.56	170.83	112.00
hsa-miR-30a-3p	140.34	243.29	104.45
hsa-miR-2110	220.68	96.65	169.49
hsa-miR-374a-5p	212.04	78.21	193.40
hsa-miR-22-5p	100.17	185.67	196.81
hsa-miR-532-3p	267.97	127.62	81.40
hsa-miR-369-3p	104.24	262.30	107.30
hsa-miR-130b-5p	105.76	188.55	164.93
hsa-miR-618	175.42	216.93	55.93
hsa-miR-450a-5p	69.15	223.26	151.70
hsa-miR-16-2-3p	108.81	181.20	153.12
hsa-miR-379-5p	69.66	165.79	166.50
hsa-miR-30b-5p	213.05	157.29	31.45
hsa-miR-382-5p	111.36	145.34	133.91
hsa-miR-497-5p	173.39	107.02	63.33
hsa-miR-192-5p	163.22	118.40	58.63
hsa-miR-152-3p	109.32	166.37	58.49
hsa-miR-204-3p	43.73	178.32	100.90
hsa-miR-320c	107.29	90.60	124.95
hsa-miR-3614-5p	194.24	97.66	29.32
hsa-miR-200c-3p	47.80	121.71	147.15
hsa-miR-194-5p	212.54	96.51	6.12
hsa-miR-205-5p	160.17	71.59	79.55
hsa-miR-598-3p	129.66	52.72	115.13
hsa-miR-651-5p	111.36	62.37	104.88
hsa-miR-340-3p	74.75	99.39	102.60
hsa-miR-378a-5p	132.71	50.99	76.56
hsa-miR-450a-1-3p	117.97	25.78	114.56
hsa-miR-548e-3p	9.66	170.11	73.72

hsa-miR-181a-3p	138.81	81.96	31.31
hsa-miR-335-3p	133.22	50.70	67.74
hsa-miR-664a-5p	17.29	106.73	124.38
hsa-miR-625-5p	169.83	36.73	38.42
hsa-miR-450a-2-3p	71.19	99.53	72.58
hsa-miR-23a-5p	33.05	78.21	119.11
hsa-miR-582-5p	69.15	44.65	110.72
hsa-miR-191-3p	146.44	53.73	12.38
hsa-miR-1912-3p	26.95	106.16	55.64
hsa-miR-576-5p	52.88	67.41	59.20
hsa-miR-323a-3p	42.71	33.42	91.65
hsa-miR-942-5p	60.00	21.32	85.67
hsa-miR-181c-5p	62.54	63.38	38.42
hsa-miR-493-5p	61.02	46.67	49.38
hsa-miR-200a-3p	19.83	35.00	97.20
hsa-miR-1270	67.12	59.06	21.77
hsa-miR-29b-3p	80.34	37.02	29.03
hsa-miR-9985	40.68	63.23	40.13
hsa-miR-574-5p	30.51	64.53	41.41
hsa-miR-425-3p	46.27	51.13	38.57
hsa-miR-139-5p	49.83	36.30	12.95
hsa-miR-873-5p	60.00	9.22	29.03
hsa-miR-129-5p	16.78	38.89	35.86
hsa-miR-146b-3p	28.47	4.03	52.94
hsa-miR-12136	8.64	42.49	27.61
hsa-miR-329-3p	32.03	31.98	3.42
hsa-miR-10b-3p	16.78	30.10	16.79
hsa-miR-365b-3p	17.80	28.23	5.83
hsa-miR-365a-3p	19.32	24.49	6.12
hsa-miR-664a-3p	18.31	21.89	9.39
hsa-miR-941	11.69	9.65	3.42

Table S3. The complete list of the miRNAs isolated by each method. Abbreviations: mirVana PARIS Kit from Ambion (PAR), Total Exosome Isolation Reagent from Invitrogen (INV), Plasma/Serum RNA Purification Kit from Norgen (NOR), and the fractions 3 (F03) and 9 (F09) of the SEC.

Methods	Common miRNAs	miRNAs
F03 F09 INV NOR PAR	48	hsa-miR-30d-5p hsa-let-7i-5p hsa-miR-23b-3p hsa-miR-23a-3p hsa-miR-143-3p hsa-miR-125a-5p hsa-miR-10b-5p hsa-miR-423-3p hsa-miR-146a-5p hsa-miR-10a-5p hsa-miR-99a-5p hsa-miR-146b-5p hsa-miR-24-3p hsa-miR-15b-5p hsa-miR-223-3p hsa-miR-451a hsa-miR-150-5p hsa-miR-199b-5p hsa-let-7b-5p hsa-let-7f-5p hsa-miR-335-5p hsa-miR-26a-5p hsa-miR-186-5p hsa-miR-93-5p hsa-let-7a-5p hsa-miR-92a-3p hsa-let-7c-5p hsa-miR-203a-3p hsa-miR-340-5p hsa-miR-148a-3p hsa-miR-25-3p hsa-miR-204-5p hsa-miR-423-5p hsa-miR-425-5p hsa-miR-98-5p hsa-miR-3184-3p hsa-miR-29a-3p hsa-let-7g-5p hsa-miR-27b-3p hsa-miR-21-5p hsa-let-7d-3p hsa-miR-3529-3p hsa-miR-100-5p hsa-miR-22-3p hsa-miR-16-5p hsa-miR-320a-3p hsa-miR-27a-3p hsa-miR-191-5p
F03 INV NOR PAR	12	hsa-miR-92b-3p hsa-miR-99b-5p hsa-let-7e-5p hsa-miR-199a-3p hsa-miR-30c-5p hsa-miR-30a-5p hsa-miR-199b-3p hsa-miR-122-5p hsa-miR-183-5p hsa-miR-15a-5p hsa-miR-1911-5p hsa-miR-142-3p
F09 INV NOR PAR	18	hsa-miR-185-5p hsa-miR-652-3p hsa-miR-3613-5p hsa-miR-193a-5p hsa-miR-28-3p hsa-miR-378a-3p hsa-miR-142-5p hsa-miR-28-5p hsa-miR-128-3p hsa-miR-345-5p hsa-miR-19b-3p hsa-miR-199a-5p hsa-let-7d-5p hsa-miR-30e-5p hsa-miR-320b hsa-miR-197-3p hsa-miR-3184-5p hsa-miR-222-3p
INV NOR PAR	74	hsa-miR-140-5p hsa-miR-361-5p hsa-miR-1298-5p hsa-miR-502-3p hsa-miR-30e-3p hsa-miR-34c-5p hsa-miR-221-3p hsa-miR-375-3p hsa-miR-17-5p hsa-miR-542-3p hsa-miR-181a-2-3p hsa-miR-335-3p hsa-miR-450a-5p hsa-miR-18a-5p hsa-miR-2110 hsa-miR-374a-5p hsa-miR-450b-5p hsa-miR-744-5p hsa-miR-196a-5p hsa-miR-150-3p hsa-miR-424-5p hsa-miR-363-3p hsa-miR-30b-5p hsa-miR-320c hsa-miR-424-3p hsa-miR-629-5p hsa-miR-340-3p hsa-miR-126-3p hsa-miR-361-3p hsa-miR-146b-3p hsa-miR-182-5p hsa-miR-769-5p hsa-miR-19a-3p hsa-miR-15b-3p hsa-miR-1301-3p hsa-miR-574-3p hsa-miR-145-3p hsa-miR-342-3p hsa-miR-29c-3p hsa-miR-483-5p hsa-miR-181a-3p hsa-miR-144-3p hsa-miR-499a-5p hsa-miR-16-2-3p hsa-miR-339-3p hsa-miR-576-5p hsa-miR-338-5p hsa-miR-130b-5p hsa-miR-618 hsa-miR-130b-3p hsa-miR-421 hsa-miR-206 hsa-miR-382-5p hsa-miR-582-3p hsa-miR-106b-3p hsa-miR-501-3p hsa-miR-574-5p hsa-miR-548e-3p hsa-miR-223-5p hsa-miR-140-3p hsa-miR-505-3p hsa-miR-484 hsa-miR-26b-5p hsa-miR-132-3p hsa-miR-155-5p hsa-miR-532-5p hsa-miR-378a-5p hsa-miR-145-5p hsa-miR-20a-5p hsa-miR-21-3p hsa-miR-148b-3p hsa-miR-500a-3p hsa-miR-503-5p hsa-miR-328-3p
F09 INV NOR	1	hsa-miR-203b-5p
INV PAR	6	hsa-miR-3614-5p hsa-miR-425-3p hsa-miR-205-5p hsa-miR-339-5p hsa-miR-9985 hsa-miR-204-3p
NOR PAR	5	hsa-miR-27a-5p hsa-miR-3173-5p hsa-miR-4443 hsa-miR-625-3p hsa-miR-495-3p

INV NOR	45	hsa-miR-3615 hsa-miR-873-5p hsa-miR-374b-5p hsa-miR-450a-2-3p hsa-miR-1270 hsa-miR-195-5p hsa-miR-1912-3p hsa-miR-532-3p hsa-miR-497-5p hsa-miR-194-5p hsa-miR-651-5p hsa-miR-493-5p hsa-miR-598-3p hsa-miR-22-5p hsa-miR-139-5p hsa-miR-486-3p hsa-miR-941 hsa-miR-323a-3p hsa-miR-129-5p hsa-miR-342-5p hsa-miR-191-3p hsa-miR-450a-1-3p hsa-miR-32-5p hsa-miR-23a-5p hsa-miR-329-3p hsa-miR-664a-5p hsa-miR-374c-3p hsa-miR-582-5p hsa-miR-192-5p hsa-miR-10b-3p hsa-miR-29b-3p hsa-miR-200c-3p hsa-miR-12136 hsa-miR-30a-3p hsa-miR-942-5p hsa-miR-181c-5p hsa-miR-200a-3p hsa-miR-106b-5p hsa-miR-379-5p hsa-miR-625-5p hsa-miR-369-3p hsa-miR-365b-3p hsa-miR-664a-3p hsa-miR-365a-3p hsa-miR-152-3p
PAR	7	hsa-miR-576-3p hsa-miR-1262 hsa-miR-3613-3p hsa-miR-1307-3p hsa-miR-4516 hsa-miR-338-3p hsa-miR-1273h-3p
INV	30	hsa-miR-656-3p hsa-miR-654-3p hsa-miR-98-3p hsa-miR-1306-5p hsa-miR-7-1-3p hsa-miR-483-3p hsa-miR-25-5p hsa-miR-6842-3p hsa-miR-548a-3p hsa-miR-487b-3p hsa-miR-877-5p hsa-miR-3679-5p hsa-miR-224-5p hsa-miR-550a-5p hsa-miR-369-5p hsa-miR-615-3p hsa-miR-485-5p hsa-miR-660-5p hsa-miR-324-3p hsa-miR-3065-5p hsa-miR-323b-3p hsa-miR-362-5p hsa-miR-432-5p hsa-miR-130a-3p hsa-miR-590-3p hsa-miR-1180-3p hsa-miR-214-5p hsa-miR-433-3p hsa-miR-34c-3p hsa-miR-3177-3p
NOR	35	hsa-miR-149-5p hsa-miR-381-3p hsa-miR-30c-1-3p hsa-miR-589-5p hsa-miR-216b-5p hsa-miR-1226-3p hsa-miR-127-3p hsa-miR-324-5p hsa-miR-1294 hsa-miR-7705 hsa-miR-3182 hsa-miR-26a-2-3p hsa-miR-126-5p hsa-miR-4742-3p hsa-miR-494-3p hsa-miR-181d-5p hsa-miR-766-3p hsa-miR-616-5p hsa-miR-10a-3p hsa-let-7i-3p hsa-miR-1284 hsa-miR-196b-5p hsa-miR-584-5p hsa-miR-32-3p hsa-miR-18a-3p hsa-miR-200b-3p hsa-miR-337-3p hsa-miR-874-3p hsa-miR-331-3p hsa-miR-20b-5p hsa-miR-409-3p hsa-miR-1249-3p hsa-miR-29b-1-5p hsa-miR-95-3p hsa-miR-511-5p
F03	0	
F09	0	

Table S4. The complete list of the pathways predicted by ConsensusPathDB considering the 9,952 target genes of the 281 miRNAs detected in our study by smallRNAseq.

Pathway name	Set size	Candidates contained	p-value	q-value	Pathway source
Axon guidance	358	276 (77.1%)	2.83e-23	6.48e-20	**Reactome**
Membrane Trafficking	582	414 (71.1%)	5.05e-22	5.78e-19	**Reactome**
Vesicle-mediated transport	620	434 (70.0%)	7.36e-21	5.62e-18	**Reactome**
Endocytosis - Homo sapiens (human)	244	189 (77.5%)	1.06e-16	6.06e-14	**KEGG**
Axon guidance - Homo sapiens (human)	175	143 (81.7%)	1.46e-16	6.7e-14	**KEGG**
Pathways in cancer - Homo sapiens (human)	526	362 (68.8%)	9.5e-16	3.63e-13	**KEGG**
Developmental Biology	620	418 (67.4%)	1.12e-15	3.67e-13	**Reactome**
Signaling by Receptor Tyrosine Kinases	423	299 (70.7%)	1.37e-15	3.92e-13	**Reactome**
Proteoglycans in cancer - Homo sapiens (human)	201	154 (76.6%)	3.81e-13	8.9e-11	**KEGG**
Disease	510	344 (67.5%)	3.88e-13	8.9e-11	**Reactome**
MAPK signaling pathway - Homo sapiens (human)	295	213 (72.2%)	6.11e-13	1.27e-10	**KEGG**
Intracellular signaling by second messengers	245	177 (72.2%)	5.14e-11	9.76e-09	**Reactome**
Neuronal System	368	252 (68.5%)	5.54e-11	9.76e-09	**Reactome**
Protein-protein interactions at synapses	88	74 (84.1%)	2.35e-10	3.84e-08	**Reactome**
Diseases of signal transduction	248	177 (71.4%)	2.64e-10	4.03e-08	**Reactome**
Cellular senescence - Homo sapiens (human)	160	121 (75.6%)	5.52e-10	7.9e-08	**KEGG**
Hippo signaling pathway - Homo sapiens (human)	154	117 (76.0%)	6.49e-10	8.75e-08	**KEGG**
Signaling pathways regulating pluripotency of stem cells - Homo sapiens (human)	139	106 (76.3%)	2.85e-09	3.63e-07	**KEGG**
Adherens junction - Homo sapiens (human)	72	61 (84.7%)	5.03e-09	6.07e-07	**KEGG**
Insulin signaling pathway - Homo sapiens (human)	137	104 (75.9%)	6.24e-09	7.15e-07	**KEGG**
PIP3 activates AKT signaling	214	152 (71.0%)	8.26e-09	9.01e-07	**Reactome**
Bacterial invasion of epithelial cells - Homo sapiens (human)	74	62 (83.8%)	9.28e-09	9.41e-07	**KEGG**
Breast cancer - Homo sapiens (human)	147	110 (74.8%)	9.44e-09	9.41e-07	**KEGG**
Thyroid hormone signaling pathway - Homo sapiens (human)	116	90 (77.6%)	1,00E-08	9.57e-07	**KEGG**
Wnt signaling pathway - Homo sapiens (human)	149	111 (74.5%)	1.24e-08	1.14e-06	**KEGG**
Intra-Golgi and retrograde Golgi-to-ER traffic	186	134 (72.0%)	1.51e-08	1.33e-06	**Reactome**

Signaling by TGF-beta family members	96	76 (79.2%)	2.94e-08	2.49e-06	**Reactome**
cAMP signaling pathway - Homo sapiens (human)	199	141 (70.9%)	3.66e-08	2.89e-06	**KEGG**
Focal adhesion - Homo sapiens (human)	199	141 (70.9%)	3.66e-08	2.89e-06	**KEGG**
Human papillomavirus infection - Homo sapiens (human)	339	225 (66.4%)	3.98e-08	3.02e-06	**KEGG**
Ras signaling pathway - Homo sapiens (human)	232	161 (69.4%)	4.09e-08	3.02e-06	**KEGG**
FoxO signaling pathway - Homo sapiens (human)	132	99 (75.0%)	4.24e-08	3.04e-06	**KEGG**
Regulation of actin cytoskeleton - Homo sapiens (human)	213	149 (70.0%)	5.79e-08	4.02e-06	**KEGG**
Signaling by VEGF	100	78 (78.0%)	6.38e-08	4.3e-06	**Reactome**
MAPK family signaling cascades	237	163 (68.8%)	8.82e-08	5.78e-06	**Reactome**
Clathrin-mediated endocytosis	138	102 (73.9%)	9.56e-08	6,00E-06	**Reactome**
Phospholipase D signaling pathway - Homo sapiens (human)	146	107 (73.3%)	9.87e-08	6,00E-06	**KEGG**
Cushing syndrome - Homo sapiens (human)	154	112 (72.7%)	9.94e-08	6,00E-06	**KEGG**
Rap1 signaling pathway - Homo sapiens (human)	206	144 (69.9%)	1.03e-07	6.08e-06	**KEGG**
Insulin resistance - Homo sapiens (human)	107	82 (76.6%)	1.21e-07	6.92e-06	**KEGG**
Melanogenesis - Homo sapiens (human)	101	78 (77.2%)	1.38e-07	7.69e-06	**KEGG**
cGMP-PKG signaling pathway - Homo sapiens (human)	163	117 (71.8%)	1.7e-07	9.24e-06	**KEGG**
EPH-Ephrin signaling	74	60 (81.1%)	1.75e-07	9.24e-06	**Reactome**
Netrin-1 signaling	41	37 (90.2%)	1.8e-07	9.24e-06	**Reactome**
Extracellular matrix organization	294	196 (66.7%)	1.81e-07	9.24e-06	**Reactome**
L1CAM interactions	103	79 (76.7%)	1.93e-07	9.62e-06	**Reactome**
Rho GTPase cycle	144	105 (72.9%)	2,00E-07	9.74e-06	**Reactome**
Gastric cancer - Homo sapiens (human)	149	108 (72.5%)	2.23e-07	1.05e-05	**KEGG**
Death Receptor Signalling	141	103 (73.0%)	2.24e-07	1.05e-05	**Reactome**
Signaling by TGF-beta Receptor Complex	67	55 (82.1%)	2.6e-07	1.19e-05	**Reactome**
Chronic myeloid leukemia - Homo sapiens (human)	76	61 (80.3%)	2.78e-07	1.25e-05	**KEGG**
Phosphatidylinositol signaling system - Homo sapiens (human)	99	76 (76.8%)	3.08e-07	1.36e-05	**KEGG**
VEGFA-VEGFR2 Pathway	92	71 (77.2%)	5.26e-07	2.27e-05	**Reactome**
Parathyroid hormone synthesis, secretion and action - Homo sapiens (human)	106	80 (75.5%)	5.34e-07	2.27e-05	**KEGG**
Signaling by NTRKs	89	69 (77.5%)	5.58e-07	2.33e-05	**Reactome**
Colorectal cancer - Homo sapiens (human)	86	67 (77.9%)	5.88e-07	2.41e-05	**KEGG**
Transport of small molecules	666	407 (61.1%)	7.3e-07	2.93e-05	**Reactome**

Antigen processing: Ubiquitination & Proteasome degradation	264	176 (66.7%)	7.6e-07	3,00E-05	**Reactome**
ErbB signaling pathway - Homo sapiens (human)	85	66 (77.6%)	8.9e-07	3.46e-05	**KEGG**
AGE-RAGE signaling pathway in diabetic complications - Homo sapiens (human)	99	75 (75.8%)	9.32e-07	3.56e-05	**KEGG**
Signaling by PDGF	54	45 (83.3%)	1.43e-06	5.39e-05	**Reactome**
mapkinase signaling pathway	57	47 (82.5%)	1.53e-06	5.58e-05	**BioCarta**
Neurexins and neuroligins	57	47 (82.5%)	1.53e-06	5.58e-05	**Reactome**
Glioma - Homo sapiens (human)	71	56 (78.9%)	2.46e-06	8.81e-05	**KEGG**
NCAM signaling for neurite out-growth	59	48 (81.4%)	2.54e-06	8.93e-05	**Reactome**
Regulation of PTEN gene transcription	62	50 (80.6%)	2.57e-06	8.93e-05	**Reactome**
Choline metabolism in cancer - Homo sapiens (human)	99	74 (74.7%)	2.67e-06	9.14e-05	**KEGG**
PTEN Regulation	96	72 (75.0%)	2.94e-06	9.92e-05	**Reactome**
AMPK signaling pathway - Homo sapiens (human)	120	87 (72.5%)	3.25e-06	0.000108	**KEGG**
PI3K-Akt signaling pathway - Homo sapiens (human)	354	226 (63.8%)	3.37e-06	0.00011	**KEGG**
Signaling by NTRK1 (TRKA)	76	59 (77.6%)	3.42e-06	0.00011	**Reactome**
Translocation of GLUT4 to the plasma membrane	32	29 (90.6%)	3.47e-06	0.00011	**Reactome**
Ephrin signaling	19	19 (100.0%)	3.96e-06	0.000124	**Reactome**
Class I MHC mediated antigen processing & presentation	324	208 (64.2%)	4.73e-06	0.000146	**Reactome**
Metabolism of carbohydrates	264	173 (65.5%)	4.82e-06	0.000147	**Reactome**
HIF-1 signaling pathway - Homo sapiens (human)	100	74 (74.0%)	5.06e-06	0.000153	**KEGG**
G alpha (12/13) signalling events	89	67 (75.3%)	5.18e-06	0.000154	**Reactome**
Oxytocin signaling pathway - Homo sapiens (human)	152	106 (69.7%)	5.79e-06	0.00017	**KEGG**
Transmission across Chemical Synapses	224	149 (66.5%)	6.34e-06	0.000181	**Reactome**
Hepatitis B - Homo sapiens (human)	144	101 (70.1%)	6.39e-06	0.000181	**KEGG**
Adrenergic signaling in cardiomyocytes - Homo sapiens (human)	144	101 (70.1%)	6.39e-06	0.000181	**KEGG**
p75 NTR receptor-mediated signalling	99	73 (73.7%)	7.28e-06	0.000203	**Reactome**
Autophagy - animal - Homo sapiens (human)	128	91 (71.1%)	7.51e-06	0.000207	**KEGG**
TGF-beta signaling pathway - Homo sapiens (human)	85	64 (75.3%)	8.33e-06	0.000227	**KEGG**
MAPK1/MAPK3 signaling	203	136 (67.0%)	9.07e-06	0.000241	**Reactome**
Endocrine and other factor-regulated calcium reabsorption - Homo sapiens (human)	47	39 (83.0%)	9.08e-06	0.000241	**KEGG**
Central carbon metabolism in cancer - Homo sapiens (human)	65	51 (78.5%)	9.15e-06	0.000241	**KEGG**
Transport to the Golgi and subsequent modification	168	115 (68.5%)	9.62e-06	0.000248	**Reactome**

keratinocyte differentiation	53	43 (81.1%)	9.8e-06	0.000248	BioCarta
Synthesis of PIPs at the plasma membrane	53	43 (81.1%)	9.8e-06	0.000248	Reactome
Golgi Associated Vesicle Biogenesis	56	45 (80.4%)	9.85e-06	0.000248	Reactome
Activation of BH3-only proteins	30	27 (90.0%)	1.06e-05	0.000262	Reactome
mcalpain and friends in cell motility	30	27 (90.0%)	1.06e-05	0.000262	BioCarta
Neurotrophin signaling pathway - Homo sapiens (human)	119	85 (71.4%)	1.11e-05	0.000267	KEGG
Glycosaminoglycan metabolism	119	85 (71.4%)	1.11e-05	0.000267	Reactome
Apelin signaling pathway - Homo sapiens (human)	137	96 (70.1%)	1.15e-05	0.000274	KEGG
Glycogen metabolism	22	21 (95.5%)	1.19e-05	0.00028	Reactome
RAF/MAP kinase cascade	197	132 (67.0%)	1.21e-05	0.000283	Reactome
Golgi-to-ER retrograde transport	116	83 (71.6%)	1.26e-05	0.000292	Reactome
Signaling by EGFR	43	36 (83.7%)	1.36e-05	0.000313	Reactome
Semaphorin interactions	64	50 (78.1%)	1.39e-05	0.000317	Reactome
role of egf receptor transactivation by gpcrs in cardiac hypertrophy	33	29 (87.9%)	1.43e-05	0.000322	BioCarta
Prostate cancer - Homo sapiens (human)	97	71 (73.2%)	1.49e-05	0.000332	KEGG
Endometrial cancer - Homo sapiens (human)	58	46 (79.3%)	1.51e-05	0.000332	KEGG
Pancreatic cancer - Homo sapiens (human)	75	57 (76.0%)	1.59e-05	0.000348	KEGG
TNF signaling pathway - Homo sapiens (human)	110	79 (71.8%)	1.62e-05	0.000351	KEGG
mTOR signaling pathway - Homo sapiens (human)	151	104 (68.9%)	1.68e-05	0.000359	KEGG
Clathrin derived vesicle budding	72	55 (76.4%)	1.73e-05	0.000363	Reactome
trans-Golgi Network Vesicle Budding	72	55 (76.4%)	1.73e-05	0.000363	Reactome
Transcriptional Regulation by TP53	374	234 (62.6%)	1.86e-05	0.000388	Reactome
Cargo recognition for clathrin-mediated endocytosis	99	72 (72.7%)	1.88e-05	0.000389	Reactome
C-type lectin receptor signaling pathway - Homo sapiens (human)	104	75 (72.1%)	2.09e-05	0.000427	KEGG
Protein processing in endoplasmic reticulum - Homo sapiens (human)	165	112 (67.9%)	2.26e-05	0.000458	KEGG
Post-translational protein modification	1383	791 (57.2%)	2.33e-05	0.000468	Reactome
Opioid Signalling	82	61 (74.4%)	2.58e-05	0.000514	Reactome
Regulation of TP53 Activity	162	110 (67.9%)	2.6e-05	0.000514	Reactome
Cell-Cell communication	124	87 (70.2%)	2.7e-05	0.000528	Reactome
Collagen biosynthesis and modifying enzymes	68	52 (76.5%)	2.78e-05	0.00054	Reactome
Glucagon signaling pathway - Homo sapiens (human)	103	74 (71.8%)	2.93e-05	0.00056	KEGG

Circadian Clock	35	30 (85.7%)	2.93e-05	0.00056	Reactome
PI Metabolism	84	62 (73.8%)	3.35e-05	0.000634	Reactome
Beta-catenin independent WNT signaling	92	67 (72.8%)	3.39e-05	0.000637	Reactome
sprouty regulation of tyrosine kinase signals	20	19 (95.0%)	4.02e-05	0.000748	BioCarta
Long-term potentiation - Homo sapiens (human)	67	51 (76.1%)	4.13e-05	0.000764	KEGG
Transcriptional regulation by RUNX2	75	56 (74.7%)	4.6e-05	0.000837	Reactome
Gastric acid secretion - Homo sapiens (human)	75	56 (74.7%)	4.6e-05	0.000837	KEGG
Fc gamma R-mediated phagocytosis - Homo sapiens (human)	91	66 (72.5%)	4.81e-05	0.000868	KEGG
Thyroid cancer - Homo sapiens (human)	37	31 (83.8%)	5.42e-05	0.000957	KEGG
RHO GTPases Activate WASPs and WAVEs	37	31 (83.8%)	5.42e-05	0.000957	Reactome
Gap junction - Homo sapiens (human)	88	64 (72.7%)	5.43e-05	0.000957	KEGG
Calcium signaling pathway - Homo sapiens (human)	186	123 (66.1%)	5.93e-05	0.00103	KEGG
Intrinsic Pathway for Apoptosis	43	35 (81.4%)	5.94e-05	0.00103	Reactome
Infectious disease	253	162 (64.0%)	6.3e-05	0.00109	Reactome
bioactive peptide induced signaling pathway	33	28 (84.8%)	8.06e-05	0.00138	BioCarta
Collagen formation	92	66 (71.7%)	8.4e-05	0.00143	Reactome
TP53 Regulates Transcription of Cell Cycle Genes	51	40 (78.4%)	8.72e-05	0.00147	Reactome
MAP kinase activation	65	49 (75.4%)	9.02e-05	0.0015	Reactome
Interleukin-17 signaling	65	49 (75.4%)	9.02e-05	0.0015	Reactome
Signaling by NOTCH	120	83 (69.2%)	9.19e-05	0.00152	Reactome
Human cytomegalovirus infection - Homo sapiens (human)	225	145 (64.4%)	9.51e-05	0.00155	KEGG
links between pyk2 and map kinases	26	23 (88.5%)	9.55e-05	0.00155	BioCarta
Longevity regulating pathway - multiple species - Homo sapiens (human)	62	47 (75.8%)	9.88e-05	0.00159	KEGG
